# Cryo-electron microscopy structure of the 70S ribosome from *Enterococcus faecalis*

**DOI:** 10.1038/s41598-020-73199-6

**Published:** 2020-10-01

**Authors:** Eileen L. Murphy, Kavindra V. Singh, Bryant Avila, Torsten Kleffmann, Steven T. Gregory, Barbara E. Murray, Kurt L. Krause, Reza Khayat, Gerwald Jogl

**Affiliations:** 1 Department of Molecular Biology, Cell Biology, and Biochemistry, Brown University, Providence, RI 02912 USA; 2 Division of Infectious Diseases, Department of Internal Medicine, University of Texas Health Science Center, Houston, TX 77030 USA; 3 Center for Antimicrobial Resistance and Microbial Genomics, University of Texas Health Science Center, Houston, TX 77030 USA; 4 Department of Chemistry and Biochemistry, The City College of New York, New York, NY 10031 USA; 5 Department of Biochemistry, University of Otago, Dunedin, 9054 New Zealand; 6 Department of Cell and Molecular Biology, The University of Rhode Island, Kingston, RI 02881 USA; 7 Department of Microbiology and Molecular Genetics, University of Texas Health Science Center, Houston, TX 77030 USA

**Keywords:** Electron microscopy, Translation, Ribosome, Structural biology

## Abstract

*Enterococcus faecalis* is a gram-positive organism responsible for serious infections in humans, but as with many bacterial pathogens, resistance has rendered a number of commonly used antibiotics ineffective. Here, we report the cryo-EM structure of the *E. faecalis* 70S ribosome to a global resolution of 2.8 Å. Structural differences are clustered in peripheral and solvent exposed regions when compared with *Escherichia coli*, whereas functional centres, including antibiotic binding sites, are similar to other bacterial ribosomes. Comparison of intersubunit conformations among five classes obtained after three-dimensional classification identifies several rotated states. Large ribosomal subunit protein bL31, which forms intersubunit bridges to the small ribosomal subunit, assumes different conformations in the five classes, revealing how contacts to the small subunit are maintained throughout intersubunit rotation. A tRNA observed in one of the five classes is positioned in a chimeric pe/E position in a rotated ribosomal state. The 70S ribosome structure of *E. faecalis* now extends our knowledge of bacterial ribosome structures and may serve as a basis for the development of novel antibiotic compounds effective against this pathogen.

## Introduction

Ribosomes are the ribonucleoprotein machines responsible for protein synthesis. As such, their nucleotide and amino acid sequences are highly conserved, particularly at functional sites responsible for the catalytic steps of polymerization. How such sequence conservation translates into conservation of three-dimensional structure has yet to be systematically examined. Due to the inherent difficulty of obtaining ribosome crystals, high-resolution structures of ribosomes from only a handful of organisms have been solved by X-ray crystallography. The high degree of sequence conservation of ribosomes and, in particular, of the functional sites that are the targets of antibiotics, provides a somewhat convincing argument that structures of ribosomes from model organisms can be used to guide the rational development of new drugs targeting ribosomes from pathogenic organisms. The development of near-atomic cryo-electron microscopy (cryo-EM) image reconstruction of ribosomes^1^ provides an opportunity to experimentally test this assertion. Recently, structures of ribosomes from *Mycobacterium tuberculosis*^2^, *Staphylococcus aureus*^3,4^, and *Mycobacterium smegmatis*^5,6^ have become available allowing an examination of the conservation of antibiotic binding sites and ribosome functional centres. Indeed, it is quite possible that visualizing ribosomes from a phylogenetically broader range of organisms will reveal structural or functional aspects of the protein synthetic apparatus not seen with the most frequently employed model systems^7^. This also raises the possibility of leveraging such differences to develop more effective and specific antimicrobial therapies.

*Enterococcus*, previously known as group D Streptococcus, is a gram-positive commensal organism that is universally found in the human gastrointestinal tract. It is an organism of low virulence but has long been recognized as a cause of endocarditis (third following staphylococci and streptococci) in the community and has proven to be an important cause of hospital-associated infections, especially infections related to the gastrointestinal tract, the urinary tract, and catheter-associated infections. Endocarditis, in particular, is associated with high mortality rates^8,9^. Historically, enterococcal endocarditis was treated with a combination of ampicillin and gentamicin but increasing resistance to (and toxicity from) this combination has led to the increased reliance on other agents including, for *Enterococcus faecium*, daptomycin, and linezolid. Unfortunately, resistance is now commonly reported to several of these antibiotics as well^10^. For example, resistance to vancomycin in *E. faecium*, known as VRE, is over 50% in some locations^11,12^.

Here, our goal was to investigate the structural conservation of the *Enterococcus faecalis* ribosome compared to *Escherichia coli*, a model organism for structure-based development of ribosome-targeting compounds. In the analysis of the *E. faecalis* 70S ribosome structure determined by cryo-electron microscopy (cryo-EM) to a global resolution of 2.8 Å, we find that structural differences are located predominantly in peripheral regions, while the organization of the functional centres and antibiotic binding sites remains conserved. Comparison of intersubunit rotations in five EM classes obtained after three-dimensional classification reveals conformational changes in ribosomal protein bL31 and the presence of a tRNA molecule in a pe/E-like conformation.

## Results

### Overall structure of the *E. faecalis* 70S ribosome

We purified 70S ribosomes from *E. faecalis* strain OG1RF and analysed the complement of ribosomal proteins in the purified 70S sample by mass-spectrometry (see Supplementary Table S1). Structure determination by cryo-EM using multi-body refinement produced molecular maps that reached 2.8 Å for the 50S subunit, 3.0 Å for the 30S body domain, and 3.2 Å for the 30S head domain by gold-standard Fourier shell correlation^13^. These maps were used to generate models for the 30S head and body domain and the 50S subunit. In addition, we used focused classification of the 30S subunit to identify five 70S conformations. These maps reach resolutions between 3.5 and 4.2 Å (see Supplementary Figs. S1–S5).

To examine 70S intersubunit motions, we compared the maps from multi-body refinement to the maps from focused classification. Motions described by eigenvector 1 of multi-body refinement include an 11-degree rotation of the 30S body with respect to the 50S subunit, and a 7.5-degree rotation of the 30S head with respect to the 30S body (see Supplementary Movie 1). Motions described by eigenvector 2 include a stationary 30S body and 23-degree rotation of the 30S head with respect to the 30S body (see Supplementary Movie 2). Motions described by eigenvector 3 include 3-degree rotations of the 30S body with respect to the 50S subunit, and 22-degree rotations of the 30S head with respect to the 30S body (see Supplementary Movie 3). Inspection of the amplitudes along eigenvector 1 suggests that the motions along this eigenvector are likely discrete, whereas motions along eigenvectors 2 and 3 are likely to be continuous (see Supplementary Fig. S6)^14–16^. Classification of the particles according to the amplitudes describing eigenvector 1 generates three classes with notable differences in the L1 stalk, rotations of the 30S body and head. Comparison of these classes with those attained using focused classification of the 30S reveals additional differences (see Supplementary Figure S7). Differences include movement in the L1 stalk, rotations in the 30S body and head. This comparison, along with the movies attained from analysis of the multi-body refinement, clearly demonstrate the *E. faecalis* ribosome to adopt a multitude of conformations.

**Figure 1:**
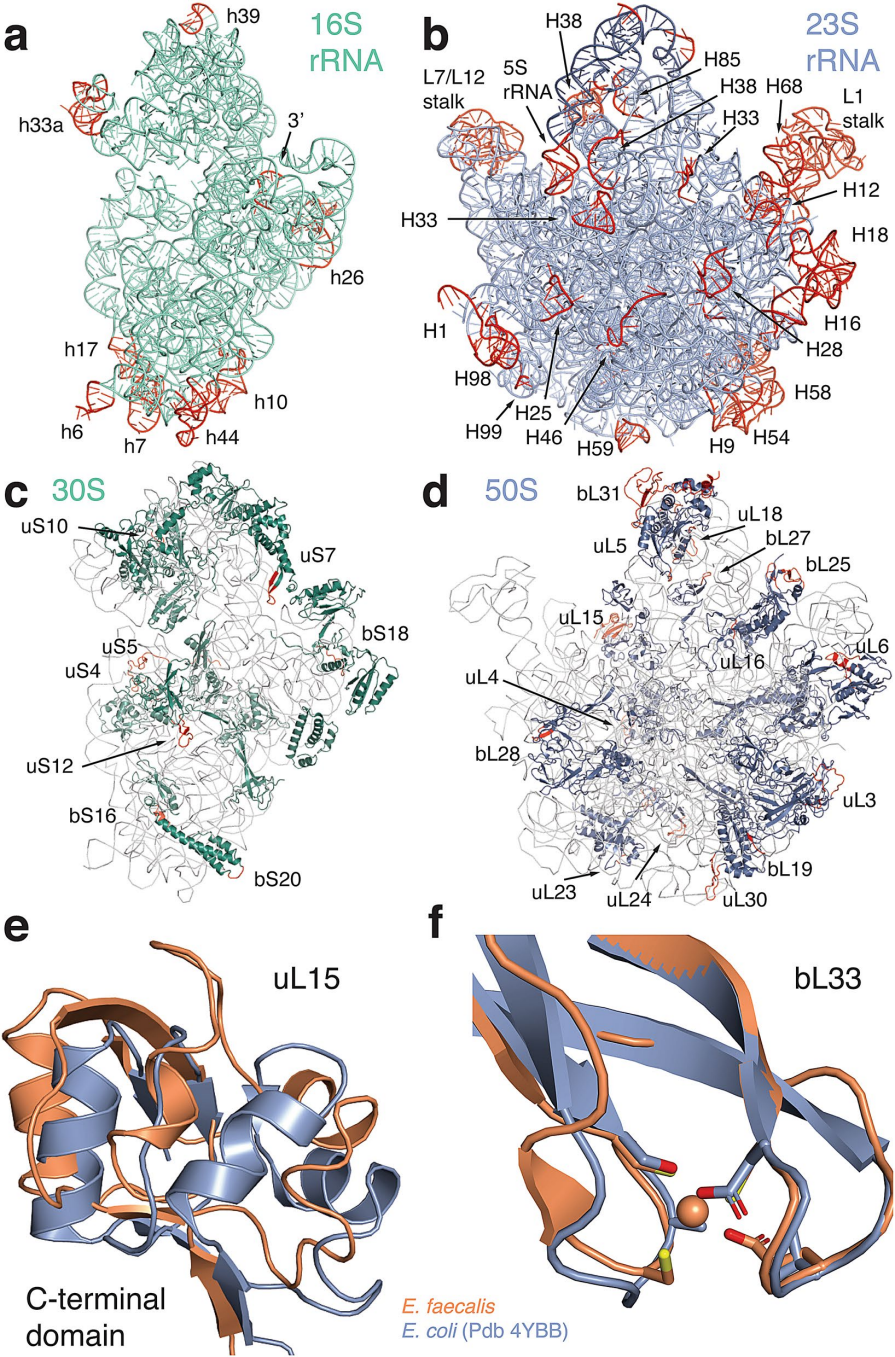
Cryo-EM structure of the *E. faecalis* ribosome, with comparison to the *E. coli* ribosome as indicated. Cartoon representations of (**a**) 16S rRNA (shown from the intersubunit interface), (**b**) 23S rRNA (shown from the solvent side), (**c**) 30S ribosomal proteins and (d) 50S ribosomal proteins. rRNA helices and ribosomal proteins with significant differences are labelled. Regions that vary from *E. coli* (PDB ID 4YBB) are coloured in red. (**e**) Conformational differences in the C-terminal domain of ribosomal protein uL15 in *E. faecalis* (orange) compared to *E. coli* (blue). (**f**) Superposition of protein bL33 in *E. faecalis* (orange) and *E. coli* (blue). Side chains for residues involved in zinc binding are shown as sticks. Zinc is shown as a sphere.

The final model of the *E. faecalis* ribosome includes 97.6% and 94% of 16S and 23S rRNA nucleotides. Ribosomal proteins that are less tightly bound and those in the L1 and L7/L12 stalks were not included in the model. The overall structure is similar to other bacterial ribosomes, as expected, and ribosome functional centres are well conserved. To identify species-specific structural differences, we compared the *E. faecalis* ribosome with a high-resolution reference structure from *E. coli* (76% sequence identity for 16S rRNA, PDB ID 4YBB^17^) and with the more closely related structure from *S. aureus* (89% sequence identity for 16S rRNA, PDB ID 5LI0^3^). In the 16S rRNA, larger structural variations are located in solvent-exposed and peripheral sections of the beak (rRNA helix h33a), the spur (h6) and the body (h7, h10 and h44, Fig. 1a). A similar pattern can be seen in the 23S rRNA, where peripheral regions show structural differences for parts of the 5S rRNA, for the functionally important L1 and L7/L12 stalks, and for 23S rRNA helices 9, 16, 18, 28, 33, 54, 58, 59, 98 and 99, among others (Fig. 1b, see Supplementary Table S4).

For ribosomal proteins, most differences are located predominantly in peripheral regions and on the solvent side of both subunits. Differences between individual proteins are located either in the N- or C-terminal regions or in loop regions that are likely to be more flexible. Despite the higher sequence conservation between *E. faecalis* and *S. aureus*, many of these regions differ among all three structures. (Fig. 1c,d, see Supplementary Tables S5).

We observed more significant structural differences for the C-terminal domain of ribosomal protein uL15 (Fig. 1e). The sequence identity for uL15 between *E. faecalis* and *E. coli* is 50%, and the rmsd between α carbons of the two protein structures is 3.4 Å. The *E. faecalis* structure contains a number of loop regions that are shifted with respect to their location in the *E. coli* structure. Some of these differences could derive from weaker electron density for protein uL15 in the *E. coli* crystal structure. However, two short helical regions that are visible in the *E. faecalis* structure but absent in the *E. coli* structure are also absent in the more closely related *S. aureus* structure (see Supplementary Fig. S9).

We also found structural differences in ribosomal protein bL33. There are three bL33 genes annotated in the *E. faecalis* genome. The cryo-EM map for bL33 is consistent with a zinc-binding motif, which is present in one of these genes (see Supplementary Fig. S9). Interestingly, the zinc-free paralogue forms of bL33 were reported in *E. coli* and *S. aureus*, whereas the zinc-binding form was modelled in *T. thermophilus*. In structural superpositions, both paralogue structures are remarkably similar to each other such that their interactions with ribosomal RNA remain conserved (Fig. 1f, see Supplementary Fig. S9).

### 70S conformations in five cryo-EM classes

**Figure 2:**
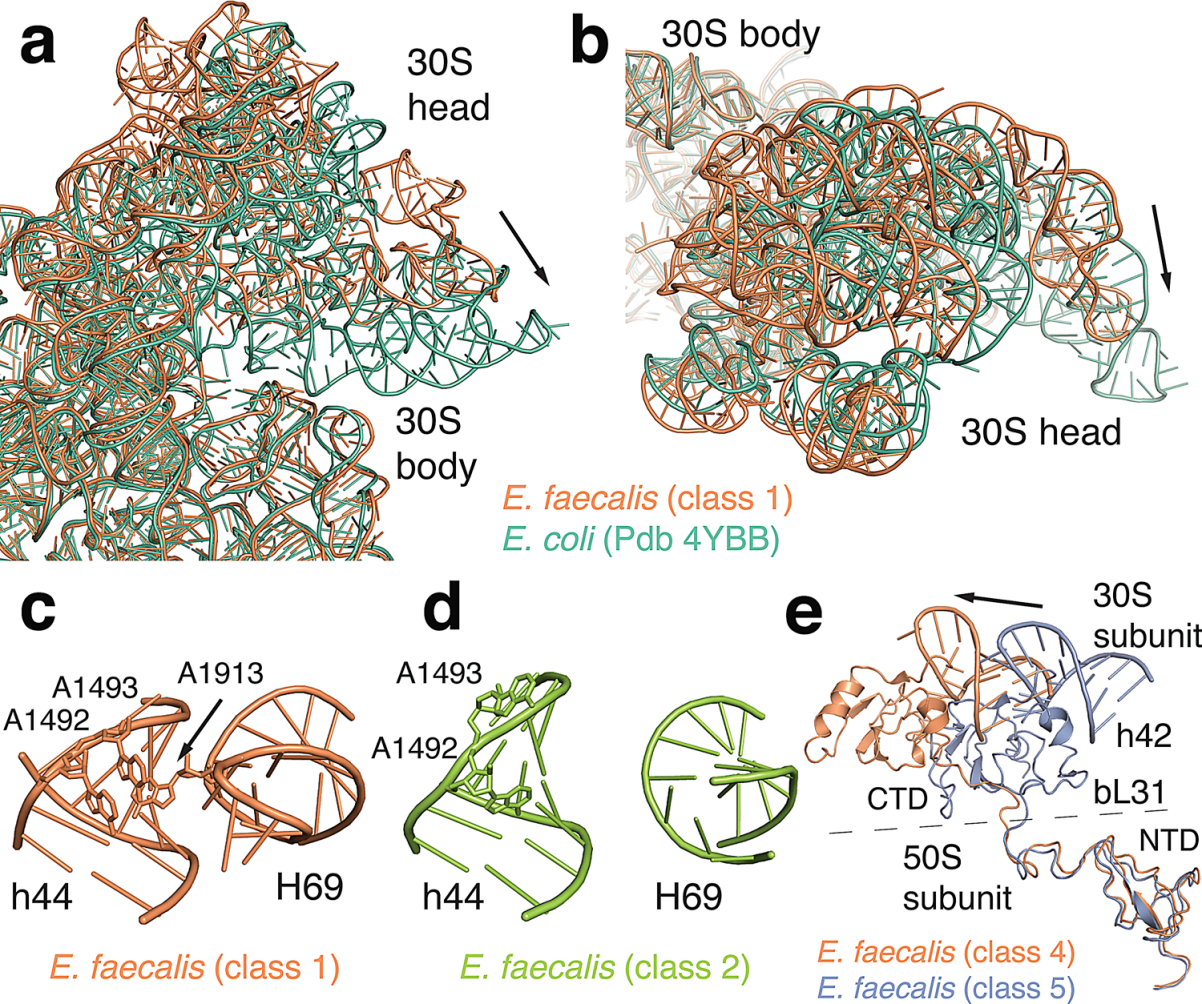
70S intersubunit rotations. (**a**,**b**) Comparison of the *E. faecalis* 30S head domain orientation (class 1, orange) with *E. coli* in a non-rotated conformation (green, PDB ID 4V51) shown from the solvent side (**a**) and from the top (**b**). Arrows indicate rotations from *E. faecalis* to *E. coli* (**c**) Intersubunit contacts between 23S helix 69 with the 30S decoding centre in class 1 compared to class 2 shown in (**d**). (**e**) Conformation of bL31 in class 4 (orange) compared with class 5 (blue) when aligned on 23S rRNA. 16S rRNA helix 42 is shown for reference.

The 70S coordinates were then fitted into the five classes attained from focused classification. The refined structures revealed large differences in the rotational state of the 30S body and head domains relative to the 50S subunit. We quantified the extent of these rotations using the Euler-Rodriguez method described by the Noller group^18^. The analysis confirms that the location of the rotation axis between the 30S body and head is conserved in *E. faecalis*. 30S body rotation ranges from 1.8° to 4.5° relative to a classical state, *E. coli* reference structure, while 30S head rotation ranges between 2.6° and 19.7° (Fig. 2, see Supplementary Table S6). 30S body rotations are all larger than in classical state ribosome structures. Even in class 4 that is closest to the classical state, the 30S body is rotated by 3.3°. The 30S head domain is significantly rotated in four of the five classes (15.5° to 19.7°) resulting in 70S conformations with large rotation of 30S body and head domains. These extensively rotated states have previously only been observed in complex with translational factors, whereas our data now capture a factor-free, rotated 70S ribosome.

We next compared how subunit rotations affect one of the central intersubunit contacts, bridge B2a between 23S helix 69 and 16S helix 44. The experimental density for the tip of helix 69 (23S nucleotides 1912–1916, *E. coli* numbering used throughout) is visible in classes 1, 4, and 5, but absent in classes 2 and 3 (Fig. 2c,d and Supplementary Fig. S10). In classes 1, 4, and 5, nucleotide A1913 in helix 69 establishes extensive contacts with the decoding centre in the small subunit. In the absence of a significant experimental density for a tRNA in the P site, this observation suggests that the contact between A1913 and the 30S decoding site does not depend on the presence of tRNAs. This interaction is also unlikely to depend on 30S head rotation, given that we observe this bridge in classes 1 and 4, which represent the largest difference in 30S head rotation (19.7° vs 2.0°). It might, however, correlate with the amount of 30S body rotation. In our structures, the molecular density for the interaction is visible for 30S body rotations between 1.8° and 3.3°, whereas no density is observed for the two structures with body rotations of 3.9° and 4.5°. In the *E. faecalis* ribosome, it is therefore possible that this bridge disengages as the 30S body rotates.

A second intersubunit bridge that changes upon subunit rotation is established by ribosomal protein bL31. This protein was not modelled in the high-resolution crystal structure of the *E. coli* ribosome but was included in a cryo-EM reconstruction of the *E. coli* ribosome in complex with EF-Tu (PDB 5AFI^19^). Bacterial genomes encode either one or two bL31 paralogs. An A form that contains a zinc-binding motif in the N-terminal domain, and a B form that does not contain this motif. The *E. faecalis* genome encodes the B type form of bL31 that does not bind zinc and that is also modelled in the *S. aureus* structure (67% sequence identity between *E. faecalis* and *S. aureus*^3^). Ribosomes from *E. coli* and *T. thermophilus* contain the A form of this protein (37% sequence identity between *E. faecalis* and *E. coli*). Comparison of the overall *E. faecalis* bL31 structure with those of *E. coli* and *S. aureus* reveals differences in length and positioning of loop regions and of the C-terminal 30S interacting region (see Supplementary Fig. S11).

The N-terminal region of bL31 is anchored to the 5S rRNA and to ribosomal protein uL5. The C-terminal part extends via a flexible linker toward the 30S subunit where it contacts ribosomal proteins uS13 and uS19^20^. Interactions with ribosomal protein uS13 establish intersubunit bridge B1c. Contacts between bL31 and ribosomal proteins uS14 and uS19 as well as contacts to 16S nucleotides A1311 and G1312 in helix 42 establish intersubunit bridge B1c^21^. The C-terminal domain of bL31 and its interactions with the 30S head domain have not been modelled in most previous structures. It is therefore still unclear how bL31 participates in 30S head rotation. In our *E. faecalis* structures, the molecular map for the flexible linker region and the C-terminal domain is well defined in the unrotated head conformation in class 4 as well as in the rotated conformation in class 5 (see Supplementary Fig. S11). The superposition of both structures confirms that the C-terminal domain retains the same interactions with the 30S head domain in both conformations (Fig. 2e). This suggests that the bL31 C-terminal domain moves as a rigid body with the 30S head domain and that the length of the flexible linker region may restrict the extent of head rotation that is possible without breaking contact with bL31.

### A tRNA bound in a chimeric pe/E orientation

**Figure 3:**
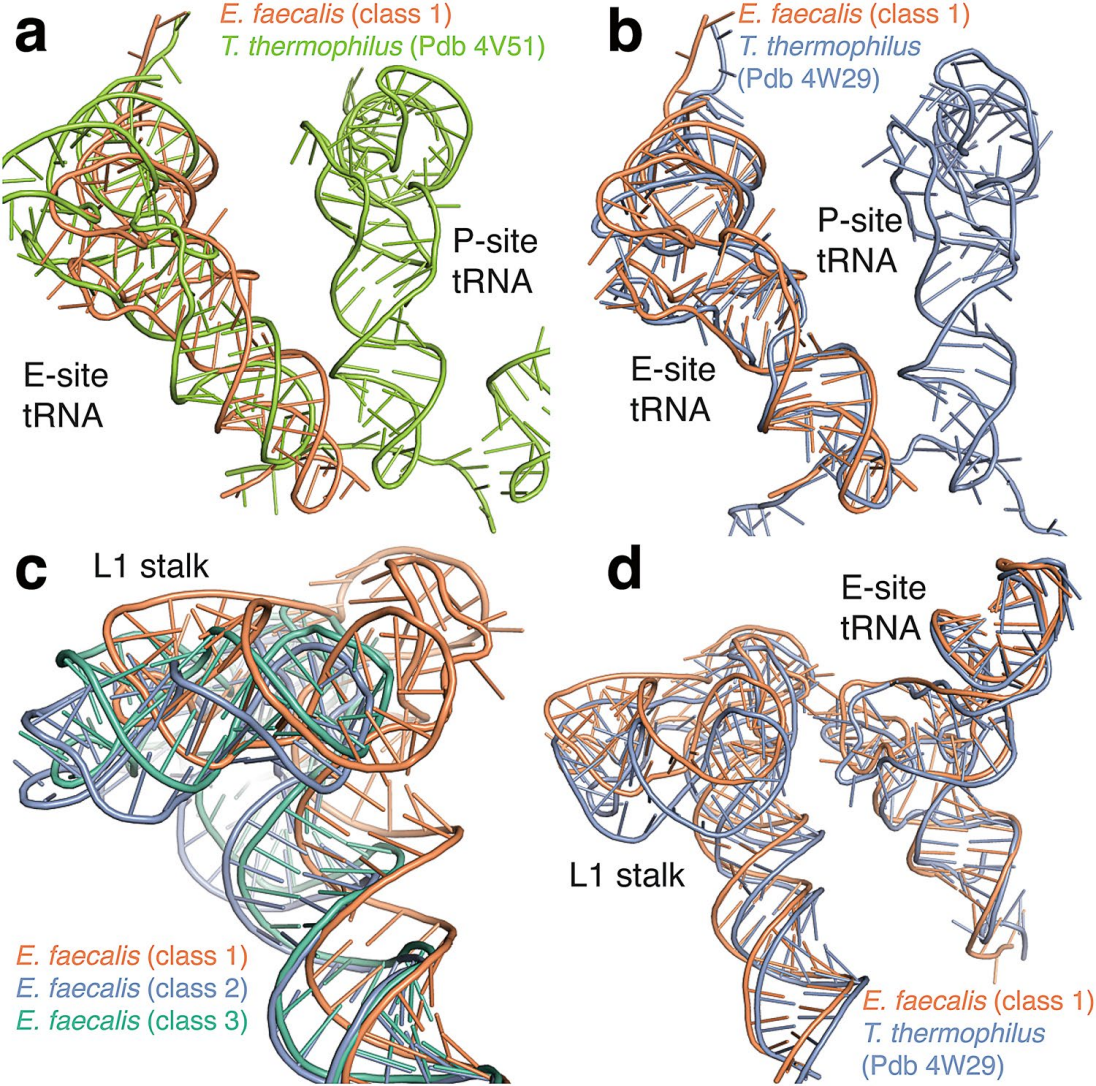
E site tRNA in a chimeric pe/E orientation. (**a**) Comparison of the tRNA position in class 1 (orange) with a classical E site position in *E. coli* (PDB ID 4V51, green) after superposition of 23S rRNA. (**b**) Comparison of the class 1 tRNA position with a chimeric pe/E tRNA in an EF-G bound 70S structure from *T. thermophilus* (PDB ID 4W29, magenta). (**c**) Comparison of L1 stalk conformations observed in three *E. faecalis* 70S structures (class 1 shown in orange, class 2 in blue, and class 3 in green). (**d**) Comparison of the L1 stalk conformation and stalk-tRNA interactions between *E. faecalis* class 1 (orange) and the chimeric *T. thermophilus* conformation in 4W29 (blue).

In class 1, the molecular map contained well-defined experimental density for a tRNA and several nucleotides of mRNA in the ribosomal E site (see Supplementary Fig. S12). This likely represents a heterogeneous mixture of tRNAs that remain bound throughout ribosome purification. We modelled an *E. coli* tRNA^Phe^ to further analyse its orientation in the E site. In class 1, the 30S body domain is only rotated by 2° whereas the 30S head is rotated by 19.7°, the largest rotation of the five *E. faecalis* structures. To examine if this rotation affects tRNA placement, we compared the structure with a classical state ribosome from *T. thermophilus* with tRNAs in the A, P, and E sites (Fig. 3a, PDB ID 4V51^22^). The comparison shows that the tRNA is not located in the classical E site orientation. Its position rather matches the conformation of a chimeric pe/E state tRNA observed in a *T. thermophilus* structure in complex with EF-G that exhibits a similar degree of 30S head and body rotation (Fig. 3b, PDB ID 4W29^23^). Chimeric hybrid orientations of tRNA have recently also been identified in *T. thermophilus* 70S ribosomes in the absence of elongation factor G^24^. One study found that a frameshift-suppressor tRNA binds in an intermediate orientation resembling a translocation intermediate^25^, and another study captured a translocation intermediate complex with two tRNAs in chimeric hybrid states^24^. It is likely that the structure we observe here resulted from the action of EF-G during the translation cycle in vivo. Our results now show that the chimeric hybrid tRNA orientation persists in bacterial ribosomes in the absence of translocation factors.

We next examined the orientation of the 23S L1 stalk in the *E. faecalis* 70S structures. The L1 stalk is a dynamic structure of the 23S rRNA that interacts with the E site tRNA. An extensive analysis of stalk rRNA conformations in ribosome structures in the PDB classified the major stalk orientations as open (vacant E site), closed (hybrid P/E tRNA), intermediate 1 (chimeric pe/E), or intermediate 2 (classical E/E)^26^. We observe three different stalk orientations in classes 1, 2, and 3 (Fig. 3c). Because the stalk interacts with the E site tRNA, we focused on the tRNA-bound class 1 structure. The stalk orientation in this structure matches closest to the intermediate 1 position observed in the chimeric hybrid state structure mentioned above (Fig. 3d). Thus, in the tRNA-bound class 1 structure, the tRNA position, the intersubunit rotation, and the orientation of the L1 stalk are all consistent with a chimeric state ribosome.

### Strong conservation of antibiotic-binding sites

Enterococcal infections are often treated by ribosome-targeting antibiotics including gentamicin, streptomycin, linezolid, quinupristin-dalfopristin, doxycycline, and tigecycline^9^. The completion of the high-resolution structure of the *E. faecalis* ribosome affords the first opportunity to inspect the binding sites for these antibiotics, which have been delineated in multiple studies of bacterial ribosomes to date^27^. Sequence conservation of these binding sites in *E. faecalis* leads to the prediction that their structure is conserved. To gain some direct insight into the structural basis of inhibition by these antibiotics, we have inspected these canonical binding sites in the enterococcal ribosome structure (see Supplementary Fig. S8).

Inspection of the binding sites for gentamicin and quinupristin confirms strong conservation in these two regions. Gentamicin binds near A1492–A1493 in the 16S helix 44, restricting motions in the ribosomal decoding centre, which contributes to misreading and interferes with translocation and ribosome recycling^28^. In Supplementary Fig. S8c, the superposed RNA backbones for the *E. coli* and *E. faec*alis structures are almost indistinguishable, indicating that antibiotic binding would occur in a similar fashion. Quinupristin is another potent anti-enterococcal agent that targets the ribosome^9^, binding in tandem with dalfopristin near the ribosomal peptidyl transferase centre. As found for gentamicin, inspection of the streptogramin binding region, including important contacts near A2062, G2057, and U746, shows high similarity in both backbone and base orientation for these two structures (see Supplementary Fig. S8d)^29^.

Linezolid, a novel oxazolidinone^9^, binds near the A-site pocket and is thought to block A-site tRNA binding as well as to interfere with initiator tRNA binding in the P site^27^. A 3–4 Å shift in the position of U2506 is noted between the superposed *E. faecalis* and *E. coli* structures, but otherwise their binding sites are similar (see Supplementary Fig. S8e)^30^. Tigecycline is a glycylcycline antibiotic that blocks delivery of aminoacylated-tRNAs to the A site by EF-Tu^27^. Within the tigecycline binding site of *E. faecalis*, U1196 in 23S helix 34 is replaced with an adenine. U1196 is known to coordinate with tigecycline and to interact indirectly with C1054^31^. Interestingly, in the *E. faecalis* ribosome, U1196 is in a loop region and now stacks with C1054 (see Supplementary Fig. S8f). While small sequence variations like this have been known to alter antibiotic binding in the ribosome^30,32^, this change in the *E. faecalis* site must not abrogate tigecycline binding because the organism remains sensitive to this antibiotic^33^.

## Discussion

The rapid technical advances of cryo-EM have opened the door for structural studies of ribosomes that are not amenable to crystallization. The cryo-EM structure of *E. faecalis* 70S ribosome presented here revealed that the architecture of functional centres and subunit interfaces remain conserved when compared with *E. coli* and *S. aureus*. Overall, we found the distribution of unique structural elements in ribosomal RNA to be similar to those observed in other bacterial ribosomes^3^, whereas their extent and conformation differ from other ribosome structures.

Analysis of ribosomal protein structures also revealed a number of changes relative to *E. coli* and *S. aureus*. In the case of uL15 and bL31, the *E. faecalis* and *S. aureus* structures cluster together as would be expected based on phylogeny. For bL33, the *E. faecalis* structure is similar to the zinc-binding paralog observed in the *T. thermophilus* ribosome. For several other proteins, we observed regions in the *E. faecalis* structure that differed from both *S. aureus* and *E. coli* (listed in Supplementary Table S4).

Our analysis of intersubunit conformations by multi-body refinement and classification revealed three classes, and traditional focused classification identified five classes. Further analysis of 70S classes identified novel intermediate rotational states that previously had only been observed in complexes with translation elongation factors. It also revealed the dynamical nature of the two domains of protein bL31 during intersubunit rotation. A complete C-terminal domain had previously been modelled in a crystal structure of the *T. thermophilus* ribosome^20^ and in cryo-EM reconstructions of the *E. coli* 70S ribosome in complexes with EF-G and EF-Tu^19^, while partial C-terminal domains have been reported in cryo-EM reconstructions of *E. coli*^34^, *M. smegmatis*^35^, and *S. aureus*^32^. Both studies with full-length bL31 only reported a single 70S conformation, leaving the question of dynamical changes in bL31 unanswered.

Several studies have probed the importance of bL31-mediated intersubunit contacts for ribosome function. Deletion of protein uS13 was shown to cause subunit association defects whereas disruption of bridge B1b (between the bL31 N-terminal domain and ribosomal protein uS13) by mutagenesis affected translation fidelity^36^. Deletion of bL31 also reduced subunit association, similar to deletion of uS13^37^. Cleavage of the last seven C-terminal residues of bL31 was sufficient to recapitulate these effects and also to compromise the formation of 100S particles in stationary phase^38^. Interestingly, there are two bL31 gene paralogs in *E. coli* which differ in the length of the flexible linker region. Dynamic exchange between the paralogs was proposed to modulate the formation of 100S particles^39^. We found in the *E. faecalis* structure that contacts between the bL31 C-terminal domain and the 30S head are maintained through a considerable range of head rotation consistent with a function in stabilizing subunit association. Further structural studies of 70S ribosomes lacking either full-length protein bL31 or carrying a C-terminally truncated protein will be necessary to better understand a potential role of bL31 in restricting intersubunit dynamics.

The presence of a bound tRNA in a rotated ribosome conformation was unexpected given the stringent protocol used to purify 70S ribosomes although this has been noted in other ribosomes studies. It is possible that the tRNA-bound 70S ribosomes were captured in a post-termination state before ribosome recycling could occur. All purification buffers contained 10 mM Mg^2+^, which inhibits subunit dissociation and may have stabilized the tRNA-bound complex. The structure we observe here is consistent with results from a recent single-molecule fluorescence study that showed that peptide release during translation termination induces a rotated ribosome conformation which is then recognized by ribosome recycling factor^40^.

Structural studies of RRF-bound ribosomes have shown that deformation of 23S helix 69 and disruption of bridge B2a initiates subunit dissociation^41,42^. Surprisingly, the structural integrity of bridge B2a appears to be dispensable for ribosome function. For example, the antibiotic capreomycin binds to H69 and capreomycin-resistance mutations have been reported that are located in H69. These include single nucleotide substitutions and deletion of a nucleotide in the tip of H69^43^. Furthermore, removal of three pseudo-uridylation sites (PSU 1911, 1915, and 1917) by deletion of the responsible pseudo-U synthase RluD causes little to no phenotype in *E. coli*^44^. While complete deletion of H69 does not affect translation significantly, ΔH69 50S subunits show subunit association defects, emphasizing the role of this bridge for stabilizing 70S ribosomes^45^. In our analysis of the *E. faecalis* ribosome, we found that bridge B2a is disordered in two 70S structures in a rotated conformation, suggesting that the integrity of this interaction is not strictly necessary once the 70S ribosome has formed. Taken together, these observations are consistent with a cooperative mechanism where the rotated state serves as substrate for RRF and also destabilizes bridge B2a to enable further deformation and disruption of this bridge to induce subunit dissociation. Further studies of mutant 70S ribosomes carrying deletions in H69 may shed further light on the role of bridge B2a in ribosome recycling.

The structural basis of action for antibiotics that target the ribosome have previously been found to be conserved across species of bacteria, regardless of their pathogenicity^2–6,27^, but to date these sites have not been studied in the enterococcal ribosome. As a result, inspection of the nucleotide sequences and binding sites of clinically relevant antibiotics such as gentamicin, quinupristin, linezolid, and tigecycline was carried out here using the *E. faecalis* structure superposed onto previously reported ribosome structures containing bound antibiotics^28–32^. As expected, the binding sites for these antibiotics were found to be generally well conserved apart from small differences in base orientation and RNA backbone, while the tigecycline site was found to contain one differing base, U1196, in 23S helix 34. Structural studies of the *E. faecalis* ribosome in the presence of these antibiotics could be carried out to directly inspect their binding modes. In addition, it is hoped that the enterococcal ribosome structure itself could have a role in developing improved antibiotics against resistant strains of this bacterial genus.

In summary, we determined the structure of the *E. faecalis* 70S ribosome to investigate its structural conservation of antibiotic-binding sites compared to the model organism *E. coli*. Both the decoding centre and the peptidyl-transferase centre structures are well conserved in *E. faecalis* when superposed with *E. coli*. However, the structural idiosyncrasies observed here may reveal potential targets for future rational drug design. Just as the binding sites for naturally occurring antibiotics and their derivatives are highly conserved, so are the mechanisms of resistance. The identification of entirely new drug binding sites could avoid known mechanisms of resistance, especially target site modification. The *E. faecalis* ribosome structure now extends our knowledge of structural variability and enables further studies exploring the sequence-structure conservation of bacterial ribosomes.

## Methods

### Isolation of ribosomes

*E. faecalis* strain OG1RF^46,47^ was streaked onto Enterococcosel Agar (EA) (Becton Dickinson) and incubated overnight. From EA plates, 20 ml of brain heart infusion broth (BHIB) was inoculated, grown ~ 6 h and 5 ml of this culture was used to inoculate 9 l of BHIB medium. Cells were grown for ~ 14 h at 37 °C to an OD_600_ of 1, harvested, washed once with saline, and stored at − 80 °C. The frozen pellet was thawed and resuspended in 20 mM K-HEPES pH 7.5, 100 mM NH_4_Cl, 10.5 mM Mg(OAc)_2_, 0.5 mM EDTA pH 8, 6 mM 2-mercaptoethanol, 0.5 mM PMSF with addition of lysozyme (1 mg/ml), mutanolysin (5 Units/ml) and DNAse I (1,250 Units). Cells were then mixed by inverting continuously at 37 °C for ~ 1 h and lysed by sonication (50% amplitude; 4 min × 2; 5 s pulse on and 30 s pulse off) while on ice. Aliquots were taken pre-lysis and post-lysis to follow CFU/ml reduction. The cell lysate was clarified by centrifugation at 20,000 rpm for 20 min at 4 °C using a Beckmann Type 45 Ti rotor and a Beckman L8-70 M ultracentrifuge. Supernatant was loaded onto a sucrose cushion (20 mM K-HEPES pH 7.5, 1.1 M sucrose, 0.5 M KCl, 10.5 mM Mg(OAc)_2_, 0.5 mM EDTA pH 8) and centrifuged at 40,000 rpm for 21 h using a Beckman Type 45 Ti rotor. The ribosomal pellet was resuspended in 20 mM Tris-OAc pH 7.5, 10 mM MgOAc_2_, 400 mM KCl, 1.2 M (NH_4_)_2_SO_4_ at 4 °C, sterilized using a syringe filter (0.22 micron), and flash frozen at − 80 °C. All glassware was pre-treated with 0.1% DEPC sterilized water and autoclaved.

Crude ribosomes were diluted in buffer C containing 20 mM Tris-OAc, pH 7.5, 400 mM KCl, 10 mM Mg(OAc)_2_, 1.2 M (NH_4_)_2_SO_4_, and loaded onto a Toyopearl butyl 650S column. Using a reverse gradient of the same buffer without (NH_4_)_2_SO_4_, 70S peak fractions were pooled and diluted in buffer E (10 mM K-HEPES, pH 7.5, 50 mM KCl, 10 mM MgOac2, and 10 mM NH_4_Cl). Ribosomes were pelleted overnight in a Ti-45 rotor at 42,000 rpm for 21 h at 4 °C. Pellets were dissolved in buffer E with 8% sucrose, loaded onto a 12 to 40% sucrose gradient, and centrifuged in a Ti-15 zonal rotor at 27,000 rpm for 21 h at 4 °C. 70S peak fractions were pooled and diluted in buffer E without sucrose and pelleted as above. Ribosomes were resuspended in buffer G (5 mM K-HEPES pH 7.5, 50 mM KCl, 10 mM Mg(OAc)_2,_ and 10 mM NH_4_Cl), concentrated using an Amicon Ultracel concentrator (30 kDa molecular weight cutoff) to a concentration of 8.9 μM, and flash-frozen at − 80 °C.

Mass-spectrometric analysis of ribosomal proteins followed standard-protocols described in the Supplementary Materials.

### Electron microscopy and image reconstruction

Protochips grids (1.2/1.4 Au) were plasma cleaned for 30 s using a Fischione Instruments 1070 NanoClean. Samples at 100 nM in buffer G (4 μl) were applied to grid for 3 s and blotted using blot force 1, blot and drain times 3.5 and 0 s, relative humidity 100% 4 °C, and plunge frozen using FEI Mark IV. Data were collected in three sessions using one FEI Titan Krios (Supplementary Fig. S1, Table S3).

MotionCor2 was used for frame alignment and dose weighting-patch 5^48^. CTFFIND4 was used for CTF estimation of unweighted micrographs^49^. Data was processed with Relion 3.1 unless indicated^50^. 5000 particles were manually selected, extracted (440 × 440), and 2D classes generated. For each data set: featureful averages served as templates for Gautomatch particle picking, particles extracted from dose-weighted micrographs, 2D classification performed. Featureful averages used to generate ab-initio model with PRIME3D of Simple 2.5^51^. 3D classification was performed to remove bad particles. Particles from data sets were combined and auto Refine3D was performed (see Supplementary Fig. S1). The refined parameters were used to initiate three-iterations of CTF Refinement (aberration, anisotropic magnification, per-particle defocus estimation) and Refine3D with Relion—generating a 2.9 Å 70S cryo-EM map. Atomic coordinates of *Thermus thermophilus* (PDB entry 4V8X) 30S head, 30S body, and 50S were docked into reconstruction and 25 Å maps calculated using UCSF Chimera’s *molmap* function^52^. Envelopes were converted into masks using *relion_mask_create* (5-pixel hard and 2-pixel soft edge) and applied to the 2.9 Å reconstruction to create reconstructions for the 30S head, 30S body, and 50S.

Multi-body refinement was performed with Relion 3.1 with the 50S subunit as body 1, the 30S body as body 2, and the 30S head as body 3. Two independent runs of multi-body refinement were carried out to determine appropriate angle and distance search parameters for each body (body 1 is stationary, bodies 2 and 3 rotate and translate with respect to body 1). Run 1 used _rlnBodySigmaAngles of 15 and _rlnBodySigmaOffset of 3. Run 2 used _rlnBodySigmaAngles of 30 and _rlnBodySigmaOffset of 6. Both runs produced indistinguishable reconstructions with similar body motions (see Supplementary Movies 1–3 online). The multi-body refinement protocol improved the resolutions to 2.9 Å for the 50S subunit, 3.0 Å for the 30S body domain, and 3.2 Å for the 30S head domain (see Supplementary Fig. S2). Three classes from the multi-body refinement were generated according to the motions described by eigenvector 1, those with amplitudes between − 40 to − 20, − 20 to 0, and 0 to 20 (see Supplementary Fig. S6). We then used relion_project to generate maps of these sub-sets and used Relion’s Refine 3D to generate high resolution maps. The maps for the three bodies were combined into a single map using UCSF Chimera *vop maximum* function. Local resolution was estimated using the MonoRes program as implemented in XMIPP^53^.

For focused classification, a 30S mask was generated using procedure described above. The 2.9 Å Relion 3.1 data star file was converted to Frealign format using scripts offered on the Frealign website^54^. Focused classification (ten classes) was carried out with Frealign for 40 cycles -no alignment. Reconstructions were visualized using UCSF Chimera. Movement of the L1 stalk and the different ratchet states of the 30S ribosomal subunit was observed. Each of the ten classes was imported into cryoSPARC and refined to produce the highest resolution reconstruction (see Supplementary Fig. S3)^55^.

### Model building and refinement

70S structures from *S. aureus* (PDB 5LI0^56^, *E. coli* (PDB 4YBB^17^), and *T. thermophilus* (PDB 4Y4P^57^) were used as starting models. Ribosomal subunits were docked into maps using Chimera^52^ and refined with phenix.real_space_refine from the Phenix package^58^. The *E. faecalis* 70S ribosome structure was built manually from these starting structures using Coot^59^ and refined in Phenix. The final 30S subunit model included 97.6% of 16S rRNA nucleotides and 18 ribosomal proteins; the 50S model included 94% of 23S rRNA nucleotides, the 5S rRNA, and 26 ribosomal proteins (see Supplementary Table S2). The model was then placed into the molecular map of the five 3D classes and the 50S subunit and 30S body domain orientation was optimized by rigid-body refinement. The 30S head domain orientation was determined with the program Molrep from the CCPEM package^60^. An *E. coli* tRNA^Phe^ and 5 mRNA nucleotides (PDB ID 4V9K^61^) were positioned in the class 1 map. Refinement was performed with phenix.real_space_refine (version 1.17.1). Model validation was performed using Molprobity and Phenix (see Supplementary Table S3). Figures were produced with PyMOL (Schrödinger, LLC.), Chimera^52^ and ChimeraX^62^.

### Accession numbers

The accession numbers for cryo-EM maps for multi-body refinement and classes 1 to 5 are EMD-21562, EMD-0656, EMD-0657, EMD-0658, EMD-0659, and EMD-0660, respectively. Atomic coordinates have been deposited with the Protein Data bank under accession numbers 6W6P, 6O8W, 6O8X, 6O8Y, 6O8Z, and 6O90.

## Supplementary information

Supplementary Information.

Supplementary Video 1.

Supplementary Video 2.

Supplementary Video 3.
