# Supplementary material for: Cryo-electron microscopy structure of the 70S ribosome from Enterococcus faecalis
Source: Sci Rep. 2020 Oct 1;10:16301. doi: 10.1038/s41598-020-73199-6 (PMC7530986; doi:10.1038/s41598-020-73199-6)
Supplement: Supplementary file 1 — Supplementary Information. [file 41598_2020_73199_MOESM1_ESM.pdf]

SUPPLEMENTARY INFORMATION

Cryo-Electron Microscopy Structure of the 70S Ribosome from *Enterococcus faecalis*

Eileen L. Murphy[1], Kavindra V. Singh[2,3], Bryant Avila[4], Torsten Kleffmann[5], Steven T. Gregory[6], Barbara E. Murray[2,3,7], Kurt L. Krause[5*], Reza Khayat[4*], and Gerwald Jogl[1*]
[1]Department of Molecular Biology, Cell Biology, and Biochemistry, Brown University, Providence, RI 02912, USA
[2]Division of Infectious Diseases, Department of Internal Medicine, University of Texas Health Science Center, Houston, TX, 77030, USA
[3]Center for Antimicrobial Resistance and Microbial Genomics, University of Texas Health Science Center, Houston, TX, 77030, USA
[4]Department of Chemistry and Biochemistry, The City College of New York, New York, NY, 10031, USA
[5]Department of Biochemistry, University of Otago, Dunedin, 9054, New Zealand
[6]Department of Cell and Molecular Biology, The University of Rhode Island, Kingston, RI, 02881, USA
[7]Department of Microbiology and Molecular Genetics, University of Texas Health Science Center, Houston, TX, 77030, USA

Supplementary Methods

Mass spectrometry-based proteomics of purified ribosomes

Sample preparation: Purified ribosomes containing 200 µg of protein were solubilized and denatured by sonication and heating to 45 °C in a buffer containing 0.1 % SDS, 5 mM tris(2-carboxyethyl)phosphine (TCEP), 5 mM EDTA/EGTA in 40 mM Tris-base (pH~10.5, no pH adjustment). The sample was then incubated with Benzonase®, for 10 min and centrifuged for 30 min at 16,000 g. The supernatant was subjected to filter-aided sample preparation (FASP) using 0.5 ml Amicon (Ultracel-3K) ultrafiltration units, essentially following the protocol by Wiśniewski et al[1]. The protein sample was digested on filter with 5 µg of trypsin at 37 °C for 12 hours and then supplemented with additional 5 µg of trypsin and digested for another 5 hours.

After digestion the peptides were purified by solid phase extraction (SPE) on a C18 Sep Pak cartridge (Waters) and fractionated into 12 factions by in solution isoelectric focusing using a 3000 OFF-GEL-Fractionator (Agilent) following the manufacturer's instructions.

Liquid chromatography-coupled tandem mass spectrometry (LC-MS/MS): Five µl sample were loaded onto an Ultimate 3000 nanoflow uHPLC system inline coupled to a nanospray ionization source of a LTQ-Orbitrap XL mass spectrometer (Thermo Scientific). Each fraction was analysed twice by two different methods using a 90 min and 120 min LC-gradient from 5 to 45% acetonitrile. Precursor ion spectra were acquired in the Orbitrap analyser in a mass range of m/z 400 to 2000 at a resolution of 60000. The strongest 11 (short method) or 14 (long method) precursor ions per cycle were selected for collision induced dissociation MS in the ion trap analyzer. For both methods dynamic exclusion was enabled allowing for two repeat measurements within a period of 90 seconds.

Data analysis: Raw data were analysed using the ProteomeDiscoverer software version 2.3 (Thermo Scientific). Peak lists were generated using default settings. All fractions were combined and searched against an *E. faecalis* (ATCC 29212 and V583 strains) amino acid sequence database (downloaded from NCBI on 14-06-2018; 15,617 sequence entries) using the Sequest HT program. Oxidation (methionine), deamidation (asparagine, glutamine), acetylation (lysine) and mono-, di- & trimethylation (lysine) were selected as variable and carbamido-methylation (cysteine) as static amino acid side chain modifications. The Perculator node was used for the estimation of false discovery rates. Only high confidence protein identifications at a false discovery rate of less than 1% were accepted as significant identifications. We allowed for proteins that were identified with one or more significant peptide identification, because several ribosomal proteins are of low molecular weight resulting in a low number of observable tryptic fragments. However, we set a conservative score (XCorr) threshold of 3 for single peptide hits and manually assessed the peak assignment in the spectra of these peptide hits. Identified proteins are listed in Supplementary Table S1.

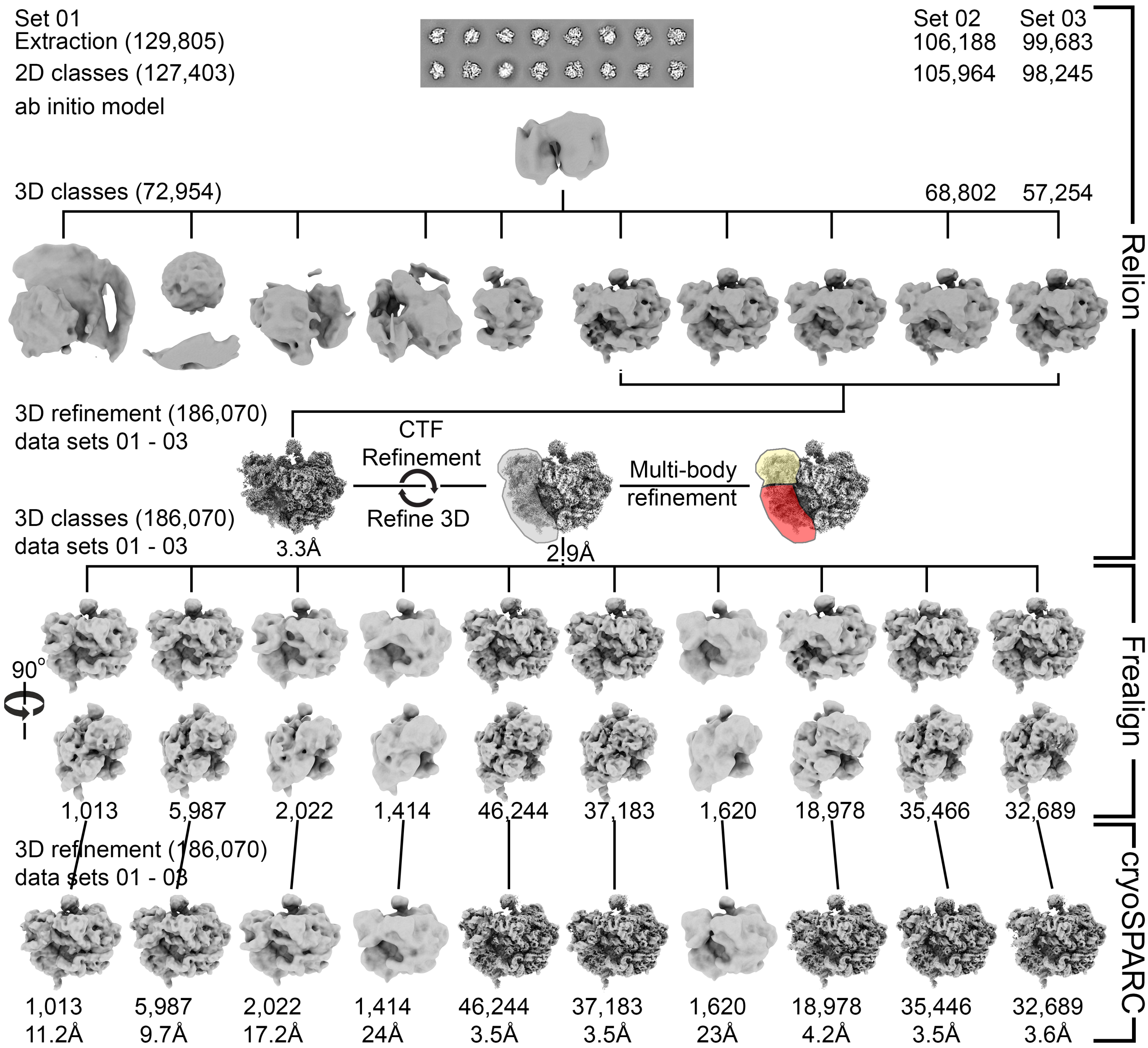

Supplementary Figure S1. Data processing overview.

Flowchart of workflow used to process cryo-EM particles extracted from dose weighted micrographs collected in three data sets (Set 01, 02, and 03). The numbers next to the processing step indicate the number of particles remaining after the executed process (e.g. 129,805 particles remained after extraction for Set 01). The best particles from the three data sets were combined to generate a single 70S cryo-EM map. The grey, red and yellow contour outlines represent the 50S, the 30S body and 30S head masks used for focused classification, and multi-body refinement. All 3D classes were refined to the highest possible resolution.

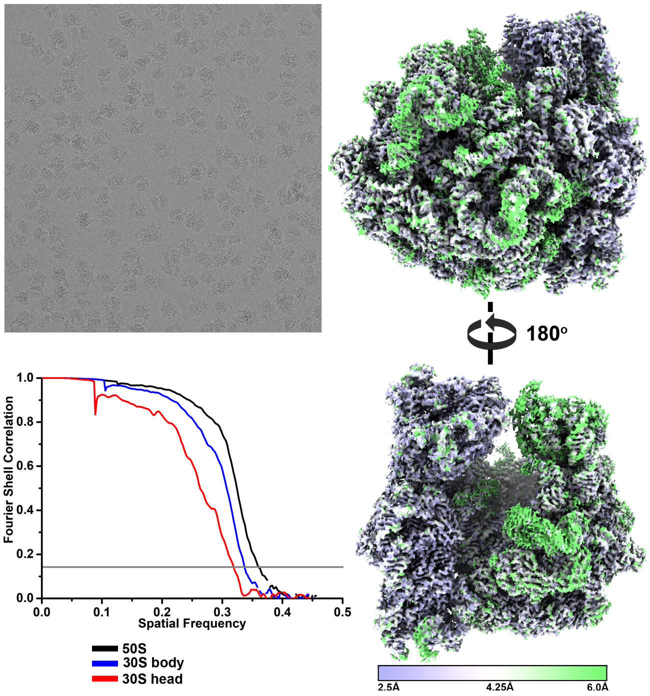

Supplementary Figure S2. Cryo-EM data collection and processing.
(a) Representative electron micrograph of *E. faecalis* 70S ribosomes. (b) Fourier Shell Correlation (FSC) curve shown for the 70S multi-body refinement. (c) Cryo-EM molecular envelope and (d) rotated by 180° coloured according to local resolution as shown.

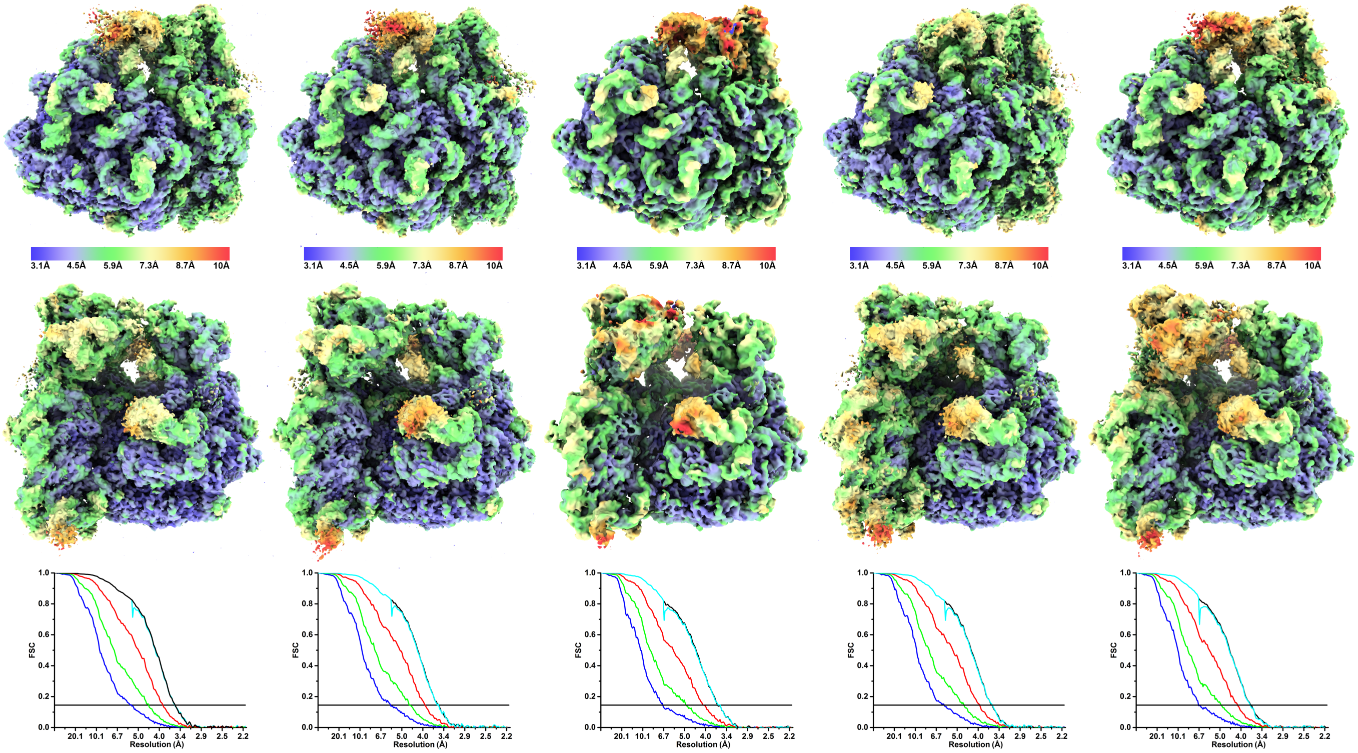

Supplementary Figure S3. Data quality for classes 1 to 5.
(a) Local resolution of image reconstructions for conformations 1 to 5. (b) Fourier Shell Correlation Curves for 70S classes 1 to 5. The coloured plots are no mask (blue), spherical (green), loose (red), tight (cyan), and corrected (black).

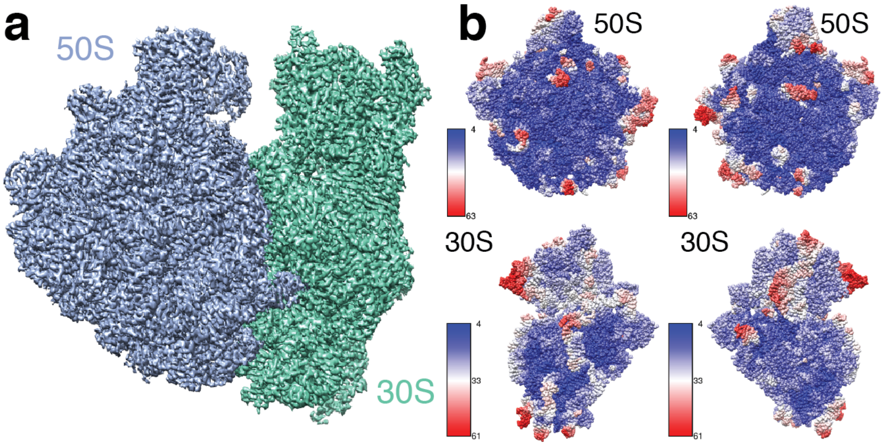

Supplementary Figure S4. Refinement.
(a) Combined multi-body refined cryo-EM map for the 70S ribosome. (b) B factor plots for (top) the 50S subunit and (bottom) the 30S subunit in two orientations, 180° apart.

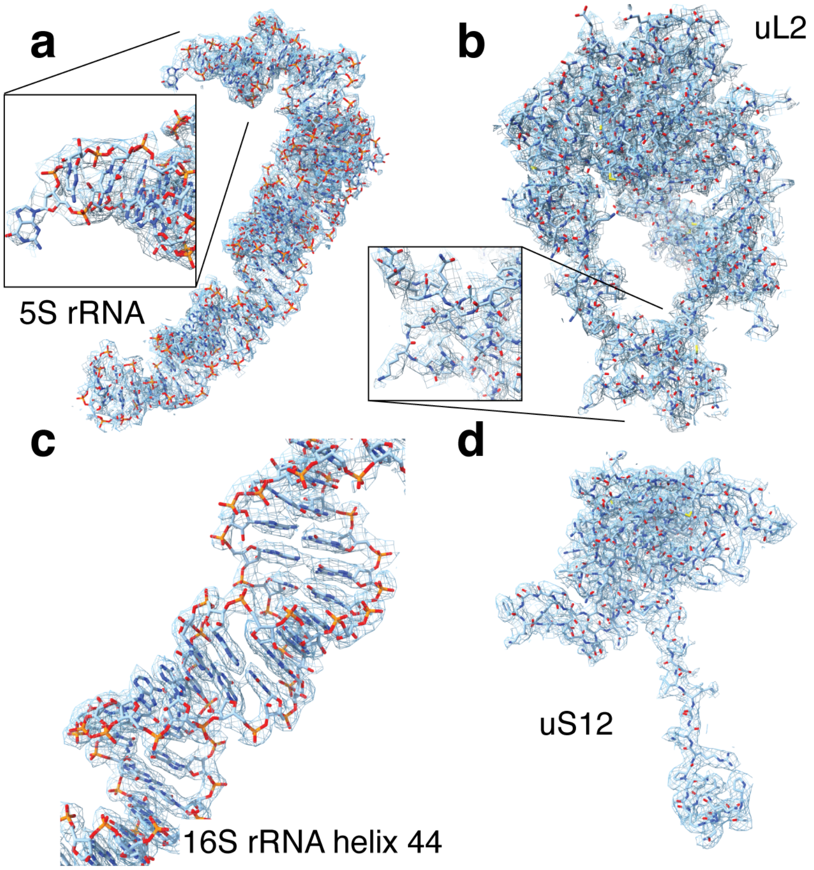

Supplementary Figure S5. Cryo-EM maps.
Cryo-EM maps for 5S rRNA (a) and ribosomal protein uL2 (b) in the 50S subunit and a section of 16S rRNA helix 44 (c) and ribosomal protein uS12 (d) are shown to illustrate the quality of the maps.

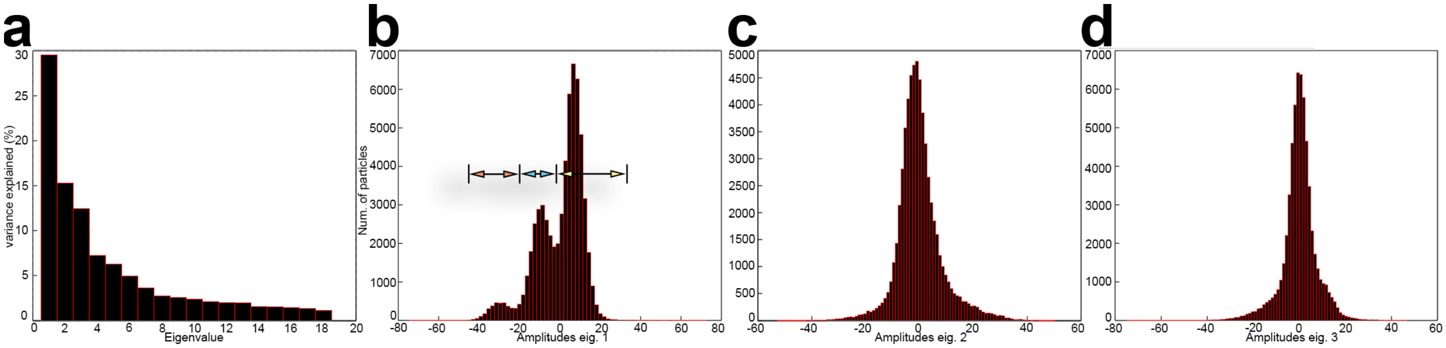

Supplementary Figure S6. Multi-body refinement

(a) Histogram of eigenvector contribution to the variance within the data. The first three eigenvectors describe 56% of the data. The first seven eigenvectors describe 75% of the data. (b) Histogram of amplitudes along eigenvector 1 shows a trimodal distributions. Particles were split into three sets to generate three classes with Relion: those with amplitudes smaller than -20 (red arrow), those with amplitudes between -20 and 0 (cyan arrow), and those with amplitudes greater than 0 (yellow arrow). (c) Histogram of amplitudes along eigenvector 2 indicating continuous motion. (d) Histogram of amplitudes along eigenvector 3 indicating continuous motion.

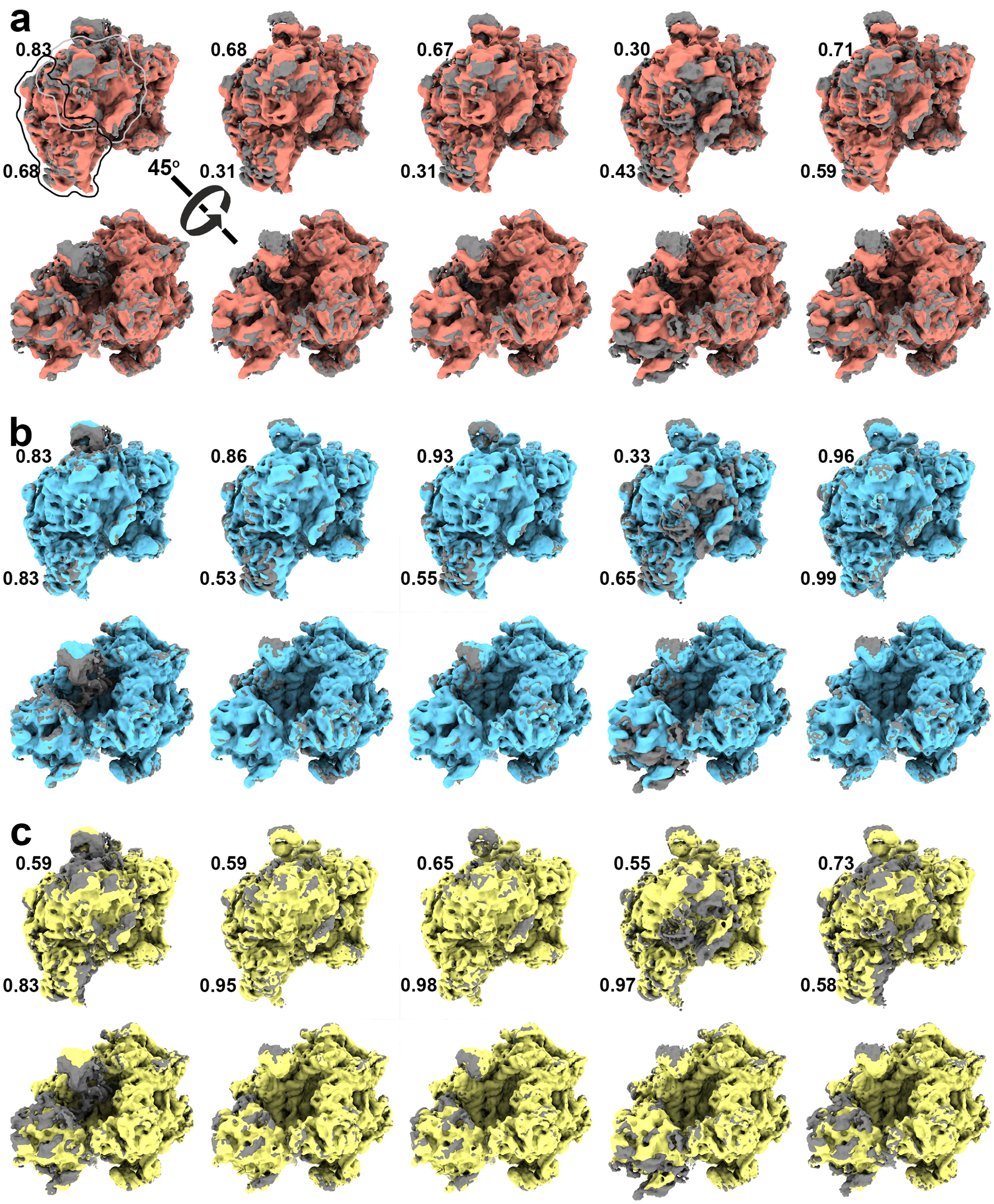

Supplementary Figure S7. Comparison of classes from multi-body and focused classification
Classes attained using multi-body refinement are compared to classes 1-5 (left to right) attained using focused classification. The multi-body classes correspond to the three peaks in the eigenvector 1 plot shown in the same colouring in Figure S6. Two orientations are shown to highlight the differences between the classes. The arrow indicates the rotation between the top and bottom images. The numbers indicate the normalized correlation coefficient for the 30S body and 30S head after the two maps were aligned according to their 50S density. The grey and black outlines in the top left image identify the 30S head and body respectively. (a) Multi-body analysis class attained from particles with amplitudes smaller than -20 in eigenvector 1 (red) compared to classes 1-5 from focused classification (grey). (b) Multi-body analysis class attained from particles with amplitudes between -20 and 0 in eigenvector 1 (blue) compared to classes 1-5 from focused classification (grey). (c) Multi-body analysis class attained from particles with amplitudes larger than 0 in eigenvector 1 (yellow) compared to classes 1-5 from focused classification (grey).

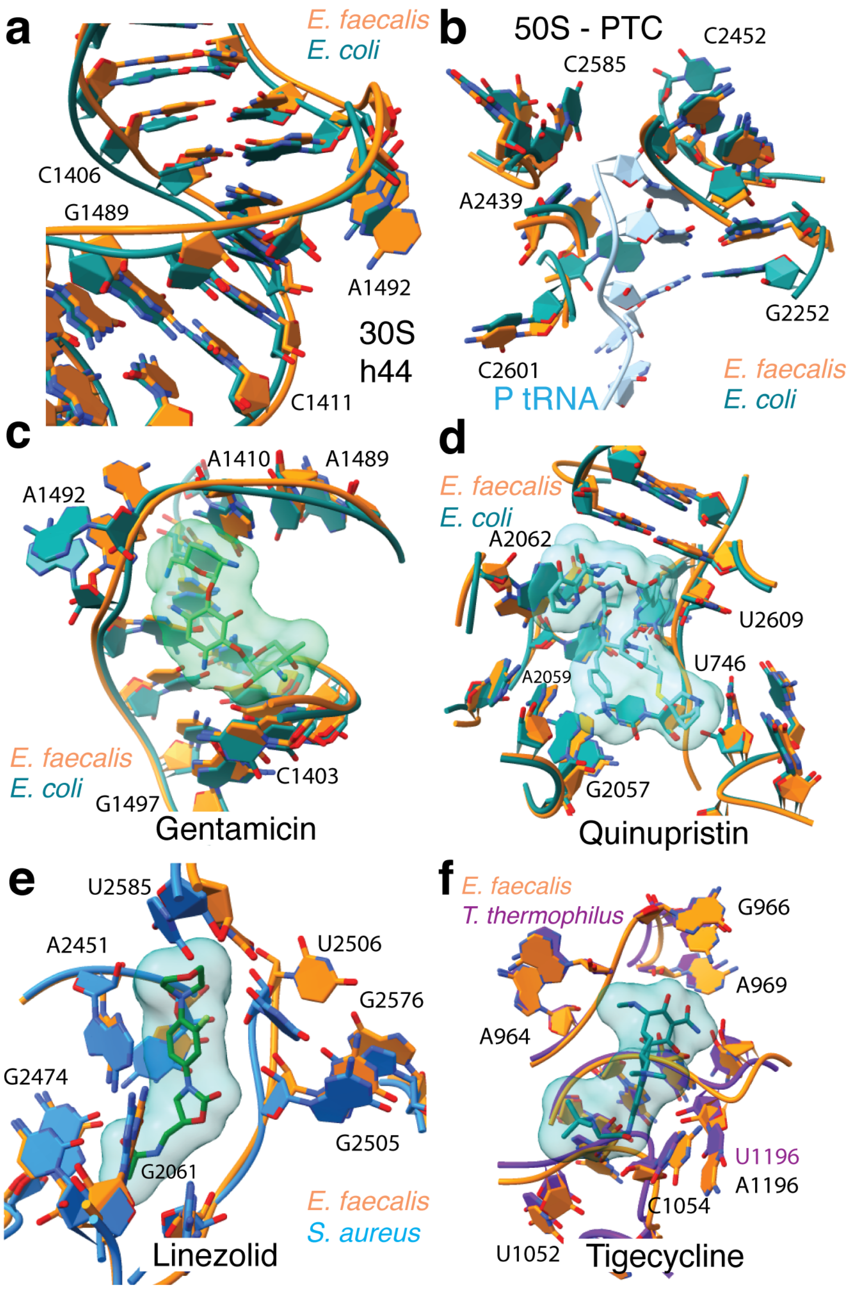

Supplementary Figure S8. Structural conservation between *E. faecalis* and *E. coli*
(a) Superposition of the 30S decoding centre comparing *E. faecalis* (orange) with *E. coli* (teal). (b) Superposition of the peptidyl-transferase centre comparing *E. faecalis* with *E. coli*. P-site tRNA (cyan) is shown for reference. (c) Comparison of the gentamicin binding site in *E. faecalis* with *E. coli* (teal, PDB 4V53[2]) (d) Comparison of the quinupristin binding site between *E. faecalis* and *E. coli* (teal, PDB 4U26[3]). (e) Comparison of the linezolid binding site in *E. faecalis* with *S. aureus* (blue, PDB 4WFA[4]). (f) Comparison of the tigecycline binding site in *E. faecalis* with *T. thermophilus* (purple, PDB 4V9B[5])

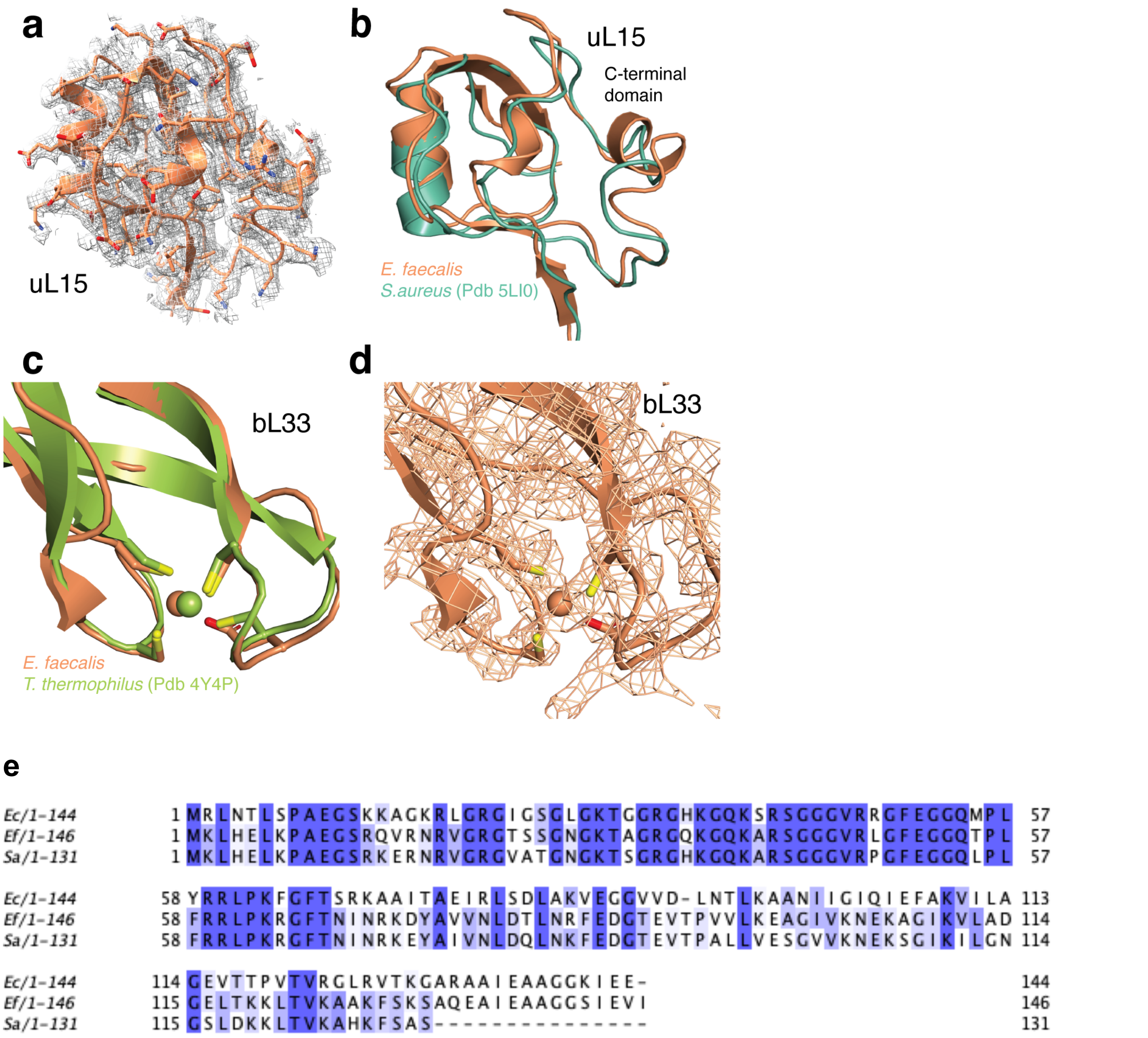

Supplementary Figure S9. Structural differences between *E. faecalis*, *S. aureus* and *T. thermophilus*. (a) Cryo-EM map for protein uL15, (b) Superposition of uL15 from *E. faecalis* and *S. aureus* (c) Superposition of bL33 from *E. faecalis* and *T. thermophilus*. (d) Cryo-EM map for ribosomal protein bL33 in class 5 (4.0 σ contour). (e) Sequence alignment for ribosomal protein L15.

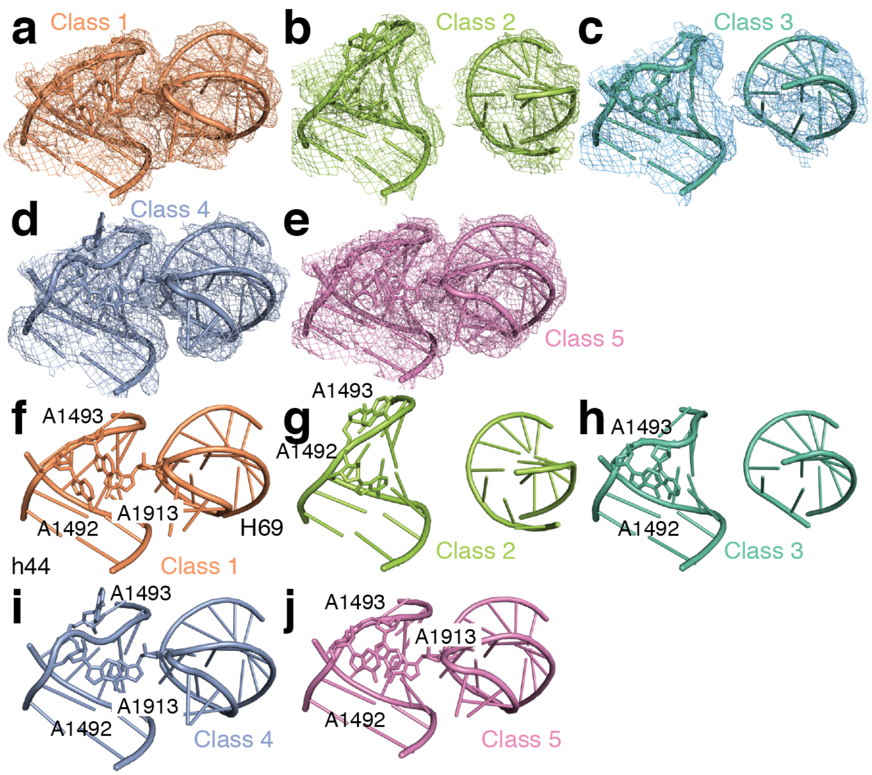

Supplementary Figure S10. Helix 69 conformation
(a-e) Cryo-EM maps (3.5 σ contour) for the bridge B2a contacts between 16S helix 44 (left) and 23S helix 69 (right) in classes 1 to 5, respectively. (f-j) cartoon representation of modelled nucleotides in each of the five classes.

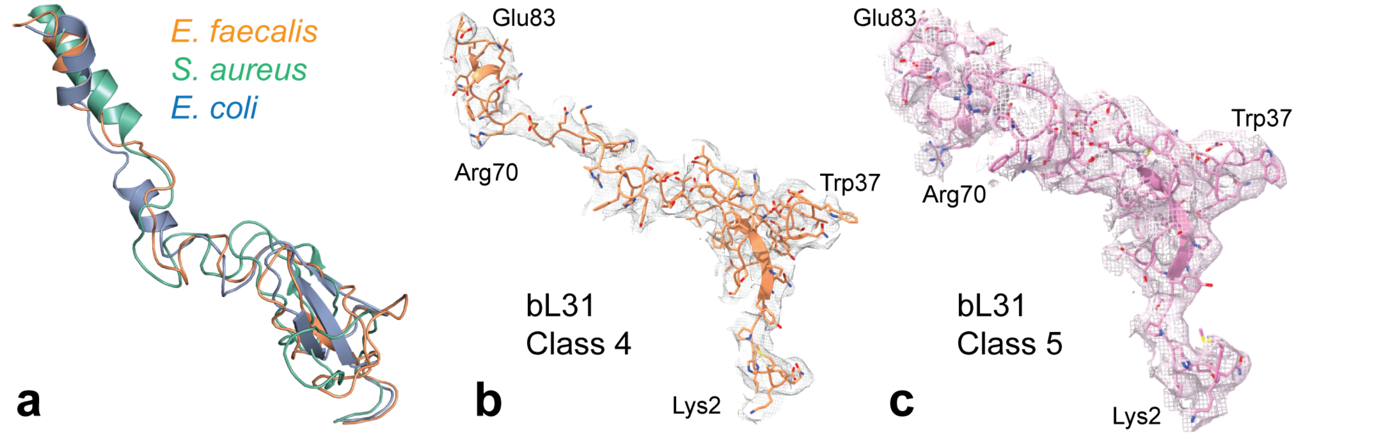

Supplementary Figure S11. Ribosomal protein bL31
(a) Superposition of ribosomal protein bL31 in *E. faecalis* (orange), *S. aureus* (green), and *E. coli* (blue). (b) Cryo-EM map for ribosomal protein bL31 in class 4 and (c) in class 5. The rmsd for Cα atoms is 8.6 Å between *E. faecalis* and *S. aureus*, and 7.6 Å between *E. faecalis* with *E. coli.*

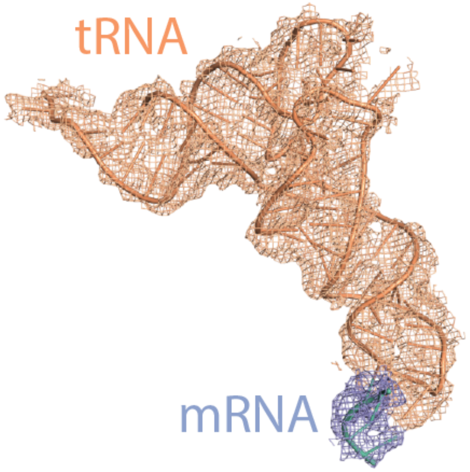

Supplementary Figure S12.
Cryo-EM map for tRNA (orange) and mRNA (blue) in class 1 (3.5 σ contour).

Supplementary Movies S1 – S3.
Movies visualize the rigid-body movement described by Relion's Multi-Body refinement (50S, 30S body, and 30S head). Supplementary Movie 1 visualizes the movements described by eigenvector 1. Supplementary Movie 2 visualizes the movements described by eigenvector 2, and Supplementary Movie 3 describes the movements described by eigenvector 3.

Table S1. Mass-spectrometry results. List of highest scoring proteins of purified *E. faecalis* ribosomes identified by mass spectrometry.

Accession (gi|number)	Protein description	Area under the curve*	emPAI**	Number of peptide spectrum matches (PSM)	Number of identified peptides***	Number of unique peptides
29342321	ribosomal protein L5 [Enterococcus faecalis V583]	1.70E+10	17011.54	1720	15	15
73621756	RecName: Full=50S ribosomal protein L31 type B	1.30E+10	9999.00	415	4	4
29342303	ribosomal protein S7 [Enterococcus faecalis V583]	2.80E+10	5622.41	852	11	11
73917096	RecName: Full=50S ribosomal protein L29	2.10E+09	2153.44	296	3	3
1145682716	30S ribosomal protein S6 [Enterococcus faecalis ATCC 29212]	1.80E+10	1777.28	545	6	6
401674297	LSU ribosomal protein L30p (L7e) [Enterococcus faecalis ATCC 29212]	2.90E+09	1777.28	324	2	2
401672703	SSU ribosomal protein S18p [Enterococcus faecalis ATCC 29212]	2.80E+09	1583.89	203	4	4
508211353	30S ribosomal protein S2 [Enterococcus faecalis V583]	2.90E+10	1332.52	1212	15	15
1145686616	50S ribosomal protein L6 [Enterococcus faecalis ATCC 29212]	1.80E+10	1210.53	1774	12	12
1145683984	50S ribosomal protein L20 [Enterococcus faecalis ATCC 29212]	1.10E+10	748.89	633	5	2
508176379	50S ribosomal protein L16 [Enterococcus faecalis V583]	7.10E+09	629.96	374	4	4
401674290	SSU ribosomal protein S11p (S14e) [Enterococcus faecalis ATCC 29212]	4.30E+09	516.95	288	7	7
29342312	ribosomal protein L2 [Enterococcus faecalis V583]	1.40E+10	407.42	1017	13	13
29377195	50S ribosomal protein L10 [Enterococcus faecalis V583]	1.40E+10	315.23	630	12	12
508175177	30S ribosomal protein S20 [Enterococcus faecalis V583]	1.20E+10	250.19	248	4	4
39931893	RecName: Full=50S ribosomal protein L19	5.70E+09	236.14	444	7	7
674290627	ribosomal protein S5 [Enterococcus faecalis ATCC 29212]	1.40E+10	192.07	755	12	12
674289263	30S ribosomal protein S13 [Enterococcus faecalis ATCC 29212]	1.70E+10	192.07	434	7	7
41017833	RecName: Full=30S ribosomal protein S4	6.50E+09	162.79	455	12	12
508215680	50S ribosomal protein L4 [Enterococcus faecalis V583]	2.10E+10	157.49	1192	8	8
29342308	ribosomal protein S10 [Enterococcus faecalis V583]	1.80E+10	132.35	326	9	9
1145687473	50S ribosomal protein L11 [Enterococcus faecalis ATCC 29212]	1.20E+10	122.29	428	6	6

508176367	50S ribosomal protein L15 [Enterococcus faecalis V583]	2.80E+10	99.00	687	8	8
508215702	50S ribosomal protein L36 [Enterococcus faecalis V583]	2.00E+09	99.00	127	1	1
508215402	50S ribosomal protein L13 [Enterococcus faecalis V583]	1.90E+10	76.43	382	6	6
488285219	MULTISPECIES: 50S ribosomal protein L7/L12 [Bacilli]	3.50E+07	67.13	391	6	1
674288509	ribosomal protein S16 [Enterococcus faecalis ATCC 29212]	1.80E+10	62.10	281	4	4
498521670	MULTISPECIES: 50S ribosomal protein L20 [Enterococcus]	4.40E+06	55.23	263	4	1
401673657	LSU ribosomal protein L1p (L10Ae) [Enterococcus faecalis ATCC 29212]	1.20E+10	45.42	1203	12	12
508215683	30S ribosomal protein S19 [Enterococcus faecalis V583]	1.50E+09	45.42	95	4	4
29343014	ribosomal protein L27 [Enterococcus faecalis V583]	2.30E+10	45.42	414	2	2
488284744	MULTISPECIES: 50S ribosomal protein L32 [Bacilli]	3.10E+09	45.42	109	1	1
488285007	MULTISPECIES: 30S ribosomal protein S8 [Bacilli]	7.00E+09	38.81	323	7	7
508169747	50S ribosomal protein L21 [Enterococcus faecalis V583]	1.20E+10	36.28	654	5	5
42559615	RecName: Full=50S ribosomal protein L3	1.80E+10	33.55	900	8	8
488313134	MULTISPECIES: 50S ribosomal protein L7/L12 [Enterococcus]	4.40E+06	30.62	384	6	1
29374862	50S ribosomal protein L24 [Enterococcus faecalis V583]	8.00E+09	30.62	255	4	4
508211776	50S ribosomal protein L33 3 [Enterococcus faecalis V583]	4.10E+09	30.62	91	1	1
29342311	ribosomal protein L23 [Enterococcus faecalis V583]	1.10E+10	26.83	566	7	7
488285020	MULTISPECIES: 50S ribosomal protein L17 [Bacilli]	1.60E+10	24.12	495	6	6
1145686611	50S ribosomal protein L14 [Enterococcus faecalis ATCC 29212]	6.00E+09	22.10	347	8	8
401674309	SSU ribosomal protein S3p (S3e) [Enterococcus faecalis ATCC 29212]	1.80E+10	21.76	506	8	8
401673527	SSU ribosomal protein S9p (S16e) [Enterococcus faecalis ATCC 29212]	1.50E+10	18.95	646	4	4
29374856	50S ribosomal protein L22 [Enterococcus faecalis V583]	5.00E+09	18.31	233	5	5
727117415	50S ribosomal protein L25 [Enterococcus faecalis]	1.70E+09	14.85	286	6	6
73621593	RecName: Full=50S ribosomal protein L18	4.50E+09	9.00	155	6	6
1145684626	30S ribosomal protein S15 [Enterococcus faecalis ATCC 29212]	2.40E+08	9.00	34	3	3
1145687236	30S ribosomal protein S21 [Enterococcus faecalis ATCC 29212]	2.20E+09	9.00	33	2	2

488286085	MULTISPECIES: ribosomal subunit interface protein [Bacilli]	2.90E+08	6.20	36	3	3
488284982	MULTISPECIES: 30S ribosomal protein S12 [Bacilli]	1.40E+09	5.00	116	2	2
401673922	Translation initiation factor 3 [Enterococcus faecalis ATCC 29212]	1.40E+07	2.73	14	4	4
259689662	unnamed protein product [Enterococcus faecalis V583]	1.40E+08	2.69	82	14	14
674289912	30S ribosomal protein S17 [Enterococcus faecalis ATCC 29212]	5.10E+08	2.16	73	3	3
674290490	ribonuclease R [Enterococcus faecalis ATCC 29212]	5.70E+07	1.54	62	14	14
674290096	CRS1 / YhbY domain protein [Enterococcus faecalis ATCC 29212]	5.10E+08	1.51	10	1	1
1145684417	30S ribosomal protein S14 2 [Enterococcus faecalis ATCC 29212]	3.30E+07	1.51	7	1	1
1130374245	glycosyl hydrolase family 25 [Enterococcus faecalis]	2.30E+07	1.29	46	10	10
1145684462	FMN-binding domain-containing protein [Enterococcus faecalis ATCC 29212]	5.60E+07	1.15	24	4	4
29374923	(3R)-hydroxymyristoyl-ACP dehydratase [Enterococcus faecalis V583]	1.20E+07	1.15	8	3	3
674290511	ribosomal protein L28 [Enterococcus faecalis ATCC 29212]	6.40E+08	1.15	18	2	2
29345119	ribosomal protein L33 [Enterococcus faecalis V583]	2.10E+07	1.15	5	1	1
29374864	30S ribosomal protein S14 [Enterococcus faecalis V583]	9.80E+06	1.15	2	1	1
1145680888	acyl carrier protein [Enterococcus faecalis ATCC 29212]	5.50E+06	1.15	5	1	1
401672587	Polyribonucleotide nucleotidyltransferase [Enterococcus faecalis ATCC 29212]	2.00E+07	1.12	55	11	11
1130381733	alpha-ketoacid dehydrogenase subunit beta [Enterococcus faecalis]	1.20E+07	0.97	17	5	5
488284961	MULTISPECIES: BMP family ABC transporter substrate-binding protein [Bacilli]	7.40E+06	0.91	19	6	5
1145683066	23S rRNA (uracil-5-)-methyltransferase RumA [Enterococcus faecalis ATCC 29212]	1.20E+07	0.78	14	7	7
696539409	MULTISPECIES: ribosome silencing factor RsfS [Bacilli]	1.20E+08	0.78	7	1	1
1145681387	hypothetical protein BWO99_13090 [Enterococcus faecalis ATCC 29212]	7.00E+07	0.73	20	4	4
488284915	MULTISPECIES: serine--tRNA ligase [Bacilli]	1.90E+07	0.59	16	4	4
1145687141	sugar ABC transporter substrate-binding protein [Enterococcus faecalis ATCC 29212]	1.40E+07	0.51	14	5	5

1103215327	dihydrolipoyllysine-residue acetyltransferase [Enterococcus faecalis]	3.50E+07	0.51	22	4	4
488284316	MULTISPECIES: foldase [Enterococcus]	2.40E+07	0.49	20	4	4
29342497	Na+/H+ antiporter [Enterococcus faecalis V583]	4.10E+05	0.47	3	2	2
488286231	MULTISPECIES: LapA family protein [Enterococcus]	2.20E+06	0.47	2	1	1
508173288	phosphoglycerate kinase [Enterococcus faecalis V583]	3.80E+06	0.45	11	4	4
508210373	pyruvate dehydrogenase (acetyl-transferring) E1 component, alpha subunit [Enterococcus faecalis V583]	4.60E+06	0.44	12	3	3
1130377631	hypothetical protein [Enterococcus faecalis]	1.40E+07	0.36	13	2	2
488285554	MULTISPECIES: ribosome-recycling factor [Enterococcus]	6.10E+06	0.36	2	2	2
1145687331	flavocytochrome c [Enterococcus faecalis ATCC 29212]	3.50E+06	0.35	8	4	4
508175743	M42 family peptidase [Enterococcus faecalis V583]	7.40E+05	0.33	6	2	2
488286090	MULTISPECIES: phosphate ABC transporter substrate-binding protein PstS family protein [Enterococcus]	1.60E+06	0.31	6	2	2
1145686991	type I glyceraldehyde-3-phosphate dehydrogenase [Enterococcus faecalis ATCC 29212]	8.20E+05	0.29	3	2	2
488320734	LytR family transcriptional regulator [Enterococcus faecalis]	2.00E+06	0.27	5	2	2
29342304	translation elongation factor G [Enterococcus faecalis V583]	2.20E+06	0.27	11	3	3
488288051	MULTISPECIES: ornithine carbamoyltransferase [Enterococcus]	7.30E+06	0.26	4	2	2
674289671	DNA-directed RNA polymerase, alpha subunit [Enterococcus faecalis ATCC 29212]	5.80E+06	0.25	4	2	2
1130394976	BMP family ABC transporter substrate-binding protein [Enterococcus faecalis]	4.20E+06	0.23	10	2	1
33112267	RecName: Full=Chaperone protein DnaK; AltName: Full=HSP70; AltName: Full=Heat shock 70 kDa protein; AltName: Full=Heat shock protein 70	1.10E+06	0.21	4	3	3
508176118	DNA-directed RNA polymerase subunit beta' [Enterococcus faecalis V583]	1.70E+06	0.21	11	7	7
1130381827	23S rRNA (guanosine(2251)-2'-O)-methyltransferase RlmB [Enterococcus faecalis]	3.20E+06	0.19	5	1	1
29376483	enolase [Enterococcus faecalis V583]	1.20E+07	0.18	8	2	2
488283803	MULTISPECIES: peptide ABC transporter substrate-binding protein [Bacilli]	8.90E+05	0.16	5	2	2

15625350	GroEL [Enterococcus faecalis ATCC 29212]	1.20E+06	0.15	4	2	2
29344963	glutamyl-aminopeptidase [Enterococcus faecalis V583]	6.30E+06	0.14	10	1	1
29342814	amino acid ABC transporter, amino acid-binding/permease protein [Enterococcus faecalis V583]	5.70E+06	0.12	5	2	2
674289719	iron-containing alcohol dehydrogenase family protein [Enterococcus faecalis ATCC 29212]	9.30E+06	0.12	5	1	1
674289455	tyrosine--tRNA ligase [Enterococcus faecalis ATCC 29212]	9.10E+06	0.10	2	1	1
1130377498	ATP-dependent helicase [Enterococcus faecalis]	1.20E+08	0.10	9	1	1
39931261	RecName: Full=Translation initiation factor IF-2	3.00E+06	0.10	6	2	2
1103215315	LysM peptidoglycan-binding domain-containing protein [Enterococcus faecalis]	4.40E+06	0.07	5	1	1
488286321	MULTISPECIES: formate acetyltransferase [Enterococcus]	5.90E+06	0.05	4	1	1

* The area under the curve is the average area of the peptide peak intensities of the strongest three peptide signals of each protein (top 3 intensity area). For proteins that were identified with three or less peptides the average area was calculated based on all identified peptides. The top 3 intensity area can be used as a rough measure for the relative abundance of proteins in the sample.

** The exponentially modified protein abundance index (emPAI) is calculated based on the number of peptide spectrum matches (PMSs) per protein normalized to the size of the protein. It can also be used as a rough measure for the relative abundance of proteins in the sample.

*** Only high confidence peptide identifications at a false discovery rate of $q \leq 0.01$ have been considered. Proteins that were identified by at least 2 high confidence peptide hits were accepted as being identified. Since several ribosomal proteins are of low molecular weight and may yield only a low number of observable peptide species upon tryptic digestion, we also considered proteins that were identified by a single peptide only. However, the spectra of those single peptide hits were evaluated manually for a correct peak assignment.

Table S2. Chain IDs and residue ranges of the *E. faecalis* 70S ribosome modelled in the multi-body refined envelope.

	Chain ID	No. residues	Modelled residues	Locus_tag	Comments
**30S subunit**					
16S rRNA	a	1566	3-71; 97-1550	OG1RF_RS11245	
bS1	-	403		OG1RF_RS06555	Not observed in EM maps.
uS2	-	261		OG1RF_RS09385	Not observed in EM maps.
uS3	c	218	2-205	OG1RF_RS00790	
uS4	d	203	2-202	OG1RF_RS11990	
uS5	e	166	3-165	OG1RF_RS00845	
bS6	f	100	3-99	OG1RF_RS00045	
uS7	g	156	2-155	OG1RF_RS00730	
uS8	h	132	2-132	OG1RF_RS00830	
uS9	i	130	3-130	OG1RF_RS12685	
uS10	j	102	4-102	OG1RF_RS00755	
uS11	k	129	13-129	OG1RF_RS00885	
uS12	l	137	2-137	OG1RF_RS00725	
uS13	m	121	2-111	OG1RF_RS00880	
uS14	n	61	2-61	OG1RF_RS00825	
uS15	o	89	2-89	OG1RF_RS11965	
bS16	p	91	2-90	OG1RF_RS07250	
uS17	q	88	4-86	OG1RF_RS00805	
bS18	r	79	13-78	OG1RF_RS00055	
uS19	s	92	4-81	OG1RF_RS00780	
bS20	t	83	2-83	OG1RF_RS09590	
bS21	-	58		OG1RF_RS09460	Not observed in EM maps.
tRNA (Class 1 only)	u	76	1-76	OG1RF-RS01180	tRNA-Phe
mRNA (Class 1 only)	v	5	15-19		
**50S subunit**					
23S rRNA	A	2915	4-920, 943-1094, 1148-1579, 1583-2114, 2204-2911	OG1RF_RS01070	
5S rRNA	B	116	1-116	OG1RF_RS01075	
uL1	-	229	-	OG1RF_RS10650	Not observed in EM maps.
uL2	C	276	2-276	OG1RF_RS00775	
uL3	D	209	2-208	OG1RF_RS00760	
uL4	E	207	2-207	OG1RF_RS00765	
uL5	F	179	2-178	OG1RF_RS00820	
uL6	G	178	2-177	OG1RF_RS00835	
uL9	-	150		OG1RF_RS00065	Not observed in EM maps.
uL10	-	166		OG1RF_RS10645	Not observed in EM maps.
uL11	-	140		OG1RF_RS10655	Not observed in EM maps.
bL12	-	122		OG1RF_RS10640	Not observed in EM maps.

uL13	K	147	1-145	OG1RF_RS12690
uL14	L	122	1-122	OG1RF_RS00810
uL15	M	146	1-146	OG1RF_RS00855
uL16	N	143	1-141	OG1RF_RS00795
bL17	O	127	3-126	OG1RF_RS00895
bL18	P	118	2-118	OG1RF_RS00840
bL19	Q	115	2-115	OG1RF_RS08025
bL20	R	119	2-119	OG1RF_RS03345
bL21	S	102	1-102	OG1RF_RS03640
uL22	T	115	4-115	OG1RF_RS00785
uL23	U	96	2-90	OG1RF_RS00770
uL24	V	103	1-101	OG1RF_RS00815
bL25 (not modelled in multi-body)	W	202	3-96	OG1RF_RS02900
bL27	X	95	18-93	OG1RF_RS03650
bL28	Y	62	2-55	OG1RF_RS12195
uL29	Z	62	2-62	OG1RF_RS00800
uL30	0	59	2-59	OG1RF_RS00850
bL31 type B (not modelled in multi-body)	1	89	1-83	OG1RF_RS04945
bL32	2	59	2-57	OG1RF_RS04035
bL33	3	49	1-49	OG1RF_RS11040
bL34	4	44	1-44	OG1RF_RS13195
bL35	5	66	2-65	OG1RF_RS03340
bL36	6	38	1-38	OG1RF_RS00875

Table S3. Data collection, refinement and validation statistics for 70S structures.

	Multi-body	Class 1	Class 2	Class 3	Class 4	Class 5
Rotational state		Chimeric	Vacant 2	Vacant 2	Classical	Vacant 3
Image reconstruction						
Detector	Gatan K2	Gatan K2	Gatan K2	Gatan K2	Gatan K2	Gatan K2
Mode	Counting	Counting	Counting	Counting	Counting	Counting
Magnification	22,500	22,500	22,500	22,500	22,500	22,500
Number of micrographs	2,905	2,905	2,905	2,905	2,905	2,905
Pixel Size (Å)	1.097	1.097	1.097	1.097	1.097	1.097
Number of frames	25	25	25	25	25	25
Total dose (e- Å-2)	34	34	34	34	34	34
Dose rate (e- Å-2 sec-1)	6.8	6.8	6.8	6.8	6.8	6.8
Exposure time (sec)	5	5	5	5	5	5
Defocus range (µm)	-0.41 to -3.6	-0.41 to -3.6	-0.41 to -3.6	-0.41 to -3.6	-0.41 to -3.6	-0.41 to -3.6
Particles extracted			335,676			
Particles used for reconstruction	186,070	35,466	32,689	18,978	37,183	46,244
FSC resolution (Å)*	50S: 2.8, 30S body: 3.0 30S head 3.2	3.53	3.68	4.14	3.49	3.49
B-factor sharpening (Å^2) †		-73.0	-73.6	-66.1	-76.1	-78.8
**Atomic modelling**						
CC (model to map fit)	0.83	0.86	0.85	0.84	0.86	0.86
B-factor average (rRNA)	18.3	141.0	170.1	191.8	143.3	140.0
B-factor average (protein)	14.4	136.2	162.5	182.3	122.9	126.7
**Model composition**						
Non-hydrogen atoms	133,554	140,020	138,301	138,301	138,498	138,498
Residues: protein	5,058	5,211	5,212	5,212	5,235	5,235
RNA	4,378	4,623	4,542	4,542	4,542	4,542
**Root mean square deviations**						
Bond lengths (Å)	0.01	0.007	0.007	0.008	0.01	0.009
Bond angles (°)	1.07	0.816	0.808	0.913	0.874	0.922
Chirality (°)	0.057	0.045	0.045	0.048	0.048	0.05
Planarity (°)	0.007	0.005	0.005	0.006	0.005	0.006
**Validation**						
Clashscore	6.42	19.15	20.95	40.67	16.93	20.01
Protein validation						
Molprobity score	2.89	3.42	3.44	3.82	3.35	3.46
Ramachandran favoured (%)	87.61	80.31	80.96	78.48	81.14	80.05
Ramachandran allowed (%)	12.07	19.26	18.75	21.31	18.53	19.58
Ramachandran outliers (%)	0.32	0.33	0.29	0.21	0.33	0.37
Rotamer outliers (%)	16.52	15.60	15.19	19.31	15.40	16.74
RNA validation						
Correct sugar puckers (%)	99.09	99.09	99.19	98.88	99.12	99.14
Backbone angle outliers (%)	0.00022	0.00005	0.00002	0.00002	0.00004	0.00003
PDB	6W6P	6O8W	6O8X	6O8Y	6O8Z	6O90
EMDB	EMD-21562	EMD-0656	EMD-0657	EMD-0658	EMD-0659	EMD-0660

* Fourier shell correlation reported by Relion 2.0 using the gold standard method at a correlation coefficient of 0.143. † The B-factor sharpening reported by Relion 2.0 during the post-refinement process.

Table S4: Regions with structural differences when comparing ribosomal RNA of *E. faecalis* (classical state; class 4) with *E. coli*. For each region, the residue range in *E. faecalis* and the corresponding range in *E. coli* is given.

	***E. faecalis* (class 4)**	***E. coli* vacant PDB ID: 4YBB**
	E. faecalis numbering	*E. coli* numbering
**16S rRNA**	70-100	75-95
	134-136	129-130
	182-213	182-192
	219-235	198-220
	468-495	452-480
	672-678	658-663
	850-867	835-851
	1015-1024	999-1008
	1037-1057	1021-1041
	1149-1156	1134-1141
	1455-1477	1440-1460
	1544-1550	1528-1534
5S rRNA	12	10
	32-36	34-38
	77-95	79-97
	115-116	117-120
23S rRNA	135-146	135-144
	181-185	180-182
	230-235	227-232
	273-322	270-285
	390-410	353-370
	582-588	543-551
	647-657	610-618
	690-695	651-655
	916-943	875-902
	965-973	924-932
	1093-1145	1053-1106
	1209-1218	1169-1180
	1241-1249	1202-1212
	1309-1310	1272-1274
	1444-1466	1409-1424
	1624-1642	1578-1599
	1524-1554	1481-1510
	1574-1588	1530-1542
	1755-1760	1712-1746
	1869-1902	1855-1887
	2113-2204	2099-2190
	2331-2337	2316-2323
	2801-2822	2786-2810
	2895-2898	2884-2888
	2901-2911	2890-2903

Table S5. Structural differences between ribosomal proteins of *E. faecalis* (multi-body model), *S. aureus* (Sa), and *E. coli* (Ec). Number of residues for each ribosomal protein and structural differences are listed for each organism.

	E. faecalis	***S. aureus* vacant PDB ID: 5LI0**	***E. coli* vacant PDB ID: 4YBB**
**30S subunit**			
bS1	403; not modelled	391; not modelled	557; not modelled
uS2	261; not modelled	255	241
uS3	218	217	233
uS4	203; loop 17-44 differs in all three structures	200	206
uS5	166; N-terminal extension differs from Sa	166; N-terminal extension	167; C-terminal 159-164 different
bS6	100	98	135; C-terminal 92-106 different, C-terminal 30 residues not modelled
uS7	156; loop 78-85 differs in all structures	156	179
uS8	132	132	130; loop 53-58 differs from others
uS9	130	132; C-terminus 118-132 differs from others	130; loop 56-61 differs from others
uS10	102; loop 91-96 differs in all structures	102; aa 1-4 modelled,	103; loop 33-36 differs from others,
uS11	129	129; N-terminal 10-16 differs from others	129
uS12	137; sequence insert 24-40 differs from Sa	136; sequence insert 24-40	124
uS13	121	121; C-terminal extension 115-120	118; loop 39-48 differs from others
uS14	61	61	101; inserted region 18-52
uS15	89	89	89; loop 16-24 differs from others
bS16	91; C-terminal additional helix 79-90	91; C-terminal additional helix 79-90	82; loop 41-48 differs from others
uS17	88;	87; C-terminal extension 85-87	84; N-terminus shorter and differs from others
bS18	79; N-terminal 13-24 differs from others	80;	75; N-terminal 1-20 not modelled
uS19	92	92; loop 66-70 differs from others; C-terminal 81-87 modelled	92
bS20	83; loop 42-45 differs in all structures	83	87; N-terminal 3-4 differs from others; C-terminal extension 84-87
bS21	58; not modelled	58	71; not modelled
**50S subunit**			
uL1	229; not modelled	230; not modelled	234
uL2	276	277	273; loop 20-30 differs from others, strand extension 165-168, loop 239-246 differs from others

uL3	209; loop 30-34 different in all structures; loop 84-93 different in all structures; loop 103-107 different in all structures;	220; insertion 58-68 differs from others; C-terminal 217-219 extension	209
uL4	207; insert 8-14; loop 128-133 different in all structures, loop 156-161 different in all structures	207; insert 8-14	201; loop 49-56 differs from others; loop 57-73 shifted relative to others
uL5	179; strand 72-75 differs from others;	179; helix 5-17 shifted relative to others; loop 21-31 differs from others; loop 40-44 differs from others, region 68-80 differs from others; loop 125-144 differs from others	179
uL6	178; loop 36-41 and loop 53-68 differ in all structures	178	177
bL7/L12	122; not modelled	122; not modelled	121; not modelled
uL9	150; not modelled	148; not modelled	149; involved in crystal contacts
uL10	166; not modelled	166; not modelled	165
uL11	140; not modelled	140; not modelled	142
uL13	147	145	142
uL14	122	122	123; loop 87-94 differs from others
uL15	146; C-terminal domain shifted	146; significant differences to other structures	144; secondary structure elements in C-terminal domain shifted
uL16	143; N-terminal extension 1-4, C-terminal extension 137-141 differs from others	144; N-terminal and C-terminal extension	136
bL17	127; inserted loop 68-84 differs from Sa	122; inserted loop 67-80	127; C-terminal extension 117-125
bL18	118; N-terminal extension 2-5 differs from Sa; region 22-29 differs in all structures, loop 46-51 differs in all structures, loop 58-68 significantly different in all structures,	119; N-terminal extension;	117
bL19	115; C-terminal 111-115 modelled,	116	115; C-terminal 111-115 modelled
bL20	119	118	118
bL21	102	102	103
uL22	115; N-terminal 4-6 modelled	117	110
uL23	96; loop 63-69 differs in all structures, C-terminal extension 87-90	91; C-terminal extension 87-90	100; N-terminal extension 1-5
uL24	103; loop 1-7 and loop 45-55 different in all structures, loop 71-78 differs from others	105	104
bL25	202; not modelled in multi-body; Class 4: loop 33-36 and region 54-71 different in all structures	217	94; region 10-25 shifted, helix 45-53 shifted,
bL27	95; loop 81-85 different in all structures	94; N-terminal region 12-26 differs from others	85; strand 36-40 shifted
bL28	62; loop 38-46 different in all structures,	62; region 10-29 different from others	78; long C-terminal extension 57-78
uL29	62	69; C-terminal extension 63-66	63
uL30	59	59	59
bL31 type B	89; not modelled in multi-body; Class 4: modelled differently from Sa	84	70; type A; not modelled

bL32	59; smaller shifts between all structures	58	57
bL33	49; Zn-bound conformation modelled	49; loop 32-41 differs from others; Zn-binding site not conserved in Sa	55; Zn-binding site not conserved in Ec
bL34	44	45	46; C-terminal extension 44-46
bL35	66	66; loop 2-9 shifted; loop 15-20 and region 23-41 differ from others; region 44-61 shifted	65
bL36	38	37	38

Table S6. 30S body and head rotations in *E. faecalis* 70S ribosome structures relative to an *E. coli* reference in the classical state (PDB ID 4V51).

Class	30S body rotation (°)	30S head rotation (°)	tRNA
1	2.0	19.7	pe/E
2	4.5	17.1	-
3	3.9	16.6	-
4	3.3	2.6	-
5	1.8	14.5	-

Supplementary References

1 Wisniewski, J. R., Zougman, A., Nagaraj, N. & Mann, M. Universal sample preparation method for proteome analysis. *Nat Methods* **6**, 359-362, doi:10.1038/nmeth.1322 (2009).

2 Borovinskaya, M. A. *et al.* Structural basis for aminoglycoside inhibition of bacterial ribosome recycling. *Nat. Struct. Mol. Biol.* **14**, 727-732, doi:10.1038/nsmb1271 (2007).

3 Noeske, J. *et al.* Synergy of streptogramin antibiotics occurs independently of their effects on translation. *Antimicrob. Agents Chemother.* **58**, 5269-5279, doi:10.1128/AAC.03389-14 (2014).

4 Eyal, Z. *et al.* Structural insights into species-specific features of the ribosome from the pathogen Staphylococcus aureus. *Proc. Natl. Acad. Sci. USA* **112**, E5805-5814, doi:10.1073/pnas.1517952112 (2015).

5 Jenner, L. *et al.* Structural basis for potent inhibitory activity of the antibiotic tigecycline during protein synthesis. *Proc. Natl. Acad. Sci. USA* **110**, 3812-3816, doi:10.1073/pnas.1216691110 (2013).
